# Transcriptomic Analysis of *Steinernema* Nematodes Highlights Metabolic Costs Associated to *Xenorhabdus* Endosymbiont Association and Rearing Conditions

**DOI:** 10.3389/fphys.2022.821845

**Published:** 2022-02-25

**Authors:** Emilie Lefoulon, John G. McMullen, S. Patricia Stock

**Affiliations:** ^1^School of Animal and Comparative Biomedical Sciences, University of Arizona, Tucson, AZ, United States; ^2^Department of Biology, Indiana University, Bloomington, IN, United States; ^3^College of Agriculture, California State University Chico, Chico, CA, United States

**Keywords:** transcriptome, *Steinernema*, *Xenorhabdus*, symbiosis, metabolism

## Abstract

Entomopathogenic nematodes of the genus *Steinernema* have a mutualistic relationship with bacteria of the genus *Xenorhabdus* and together they form an antagonist partnership against their insect hosts. The nematodes (third-stage infective juveniles, or IJs) protect the bacteria from the external environmental stressors and vector them from one insect host to another. *Xenorhabdus* produce secondary metabolites and antimicrobial compounds inside the insect that protect the cadaver from soil saprobes and scavengers. The bacteria also become the nematodes’ food, allowing them to grow and reproduce. Despite these benefits, it is yet unclear what the potential metabolic costs for *Steinernema* IJs are relative to the maintenance and vectoring of *Xenorhabdus*. In this study, we performed a comparative dual RNA-seq analysis of IJs of two nematode-bacteria partnerships: *Steinernema carpocapsae*-*Xenorhabdus nematophila* and *Steinernema. puntauvense*-*Xenorhbdus bovienii*. For each association, three conditions were studied: (1) IJs reared in the insect (*in vivo* colonized), (2) colonized IJs reared on liver-kidney agar (*in vitro* colonized), and (3) IJs depleted by the bacteria reared on liver-kidney agar (*in vitro* aposymbiotic). Our study revealed the downregulation of numerous genes involved in metabolism pathways, such as carbohydrate, amino acid, and lipid metabolism when IJs were reared *in vitro*, both colonized and without the symbiont. This downregulation appears to impact the longevity pathway, with the involvement of glycogen and trehalose metabolism, as well as arginine metabolism. Additionally, a differential expression of the venom protein known to be secreted by the nematodes was observed when both *Steinernema* species were depleted of their symbiotic partners. These results suggest *Steinernema* IJs may have a mechanism to adapt their virulence in absence of their symbionts.

## Introduction

Long term associations between organisms are largely widespread in nature. Among these, mutualistic symbiosis is characterized by reciprocal fitness advantages that often play a role in the function and development of both the hosts and the symbionts (Russell et al., 2014; Stoy et al., 2020). Across eukaryote host-microbe symbioses, nutrient exchange and nutritional interdependence appears pervasive (Douglas, 1998; Russell et al., 2014; Ortíz et al., 2020). Indeed, some microbial symbionts can provide essential metabolites (such as amino acids and vitamins) that the host requires but is unable to obtain without this partner (Hosokawa et al., 2010; Russell et al., 2014).

Soil-dwelling *Steinernema* third-stage infective juvenile (IJ) nematodes harbor endosymbiotic gamma-proteobacteria, *Xenorhabdus* (Poinar and Thomas 1965) Thomas and Poinar 1979, which they vector from one insect host to another. The bacteria reside in a specialized receptacle in the most anterior portion of their intestine (Bird and Akhurst, 1983; Flores-Lara et al., 2007). Once the IJs find a suitable insect host, they invade it through natural body openings (i.e., mouth, anus or spiracles) and release *Xenorhabdus* in the hemocoel, where the bacteria will proliferate (Stock, 2005). *Xenorhabdus* colonization induces septicemia and toxemia, resulting in insect mortality within 24–48 h of invasion (Boemare, 2002; Castagnola and Stock, 2014). *Xenorhabdus* has been demonstrated to produce toxins and virulence factors targeting the insect’s gut epithelium (Eleftherianos et al., 2018), as well as secondary metabolites active against the insect’s immune defenses and microbial competitors (Crawford et al., 2012; Shi and Bode, 2018). The environment inside the insect host created by *Xenorhabdus* allows *Steinernema* to develop and reproduce (Eleftherianos et al., 2018). Recent studies have also shown that Steinernema IJs also release venom proteins that are lethal to several insects including *Drosophila melanogaster* (Lu et al., 2017). Many of the venom proteins have conserved orthologs in vertebrate-parasitic nematodes suggesting the conserved function of these proteins in nematode parasitism (Lu et al., 2017). In addition, the nematodes feed on *Xenorhabdus* and degraded insect tissue until no resources are left. Then, the IJs re-associate with *Xenorhabdus* and abandon the insect cadaver in search of new hosts (Boemare, 2002). Several studies have shown that *Xenorhabdus* plays a key role in the nematode’s fitness (Sicard et al., 2003; Murfin et al., 2015; McMullen et al., 2017; Eleftherianos et al., 2018; Stock, 2019). Furthermore, the *Steinernema-Xenorhabdus* model has been used to study physiological and developmental aspects of beneficial symbiotic associations (Stock, 2005; Stock and Blair, 2008).

The level of dependence of this symbiotic partnership is variable among *Steinernema* species, although it is always mutualistic (Sicard et al., 2003). *Xenorhabdus* need the nematodes for their transmission and survival as they cannot survive more than few days in the soil (Morgan et al., 1997). Nevertheless, it has been demonstrated that some *Steinernema* species such as *Steinernema riobrave* Cabanillas, Poinar and Raulston, 1994 need the symbiont to reproduce while some other species are able to successfully produce progeny after depletion of *Xenorhabdus* (for example, *S. carpocapsae* and *Steinernema scapterisci* Nguyen and Smart, 1990; Sicard et al., 2003). The level of specialization of *Steinernema* nematodes with their native and non-native *Xenorhabdus* symbionts have also been investigated (Sicard et al., 2004, 2005;Murfin et al., 2015; McMullen et al., 2017). Results from these studies revealed that nematode fitness declines with the increase of the phylogenetic distance between the native and tested non-cognate bacteria suggesting that specificity of the association may impact the virulence and the longevity of the nematodes (Murfin et al., 2015; McMullen et al., 2017). Co-phylogenetic studies have also revealed no universal co-evolution pattern between *Steinernema-Xenorhabdus* and suggested horizontal transfer of symbionts (Lee and Stock, 2010). These associations appear to have evolved from different acquisition events and may represent different stages of a host-dependent adaptation (Sicard et al., 2005).

The partnership with *Xenorhabdus* brings benefits to *Steinernema*, especially which respect to insect invasion. However, the metabolic costs or gains for the IJs in relation to the carriage of *Xenorhabdus* symbiont remain poorly known. IJs are non-feeding stage with a focal function of foraging for an insect host. This role presents two constraints: the decline of energy storage and limitations imposed by the lifespan of the bacterial symbionts (Akhurst and Boemare, 1990). Different foraging behaviors have been described in *Steinernema*: ambush foragers that are sedentary and cruising foragers, which move actively to seek for an insect host (Campbell and Gaugler, 1993). Energy demands in cruisers are higher than in ambushers. For example, Lewis et al. (1995) suggested that ambush foragers may have lower metabolic rate to extend their survival. Furthermore, *S. carpocapsae* IJs which are ambushers, survive long-term dehydration periods (up to 7 months) as opposed to cruisers, such as *S. riobrave* or *Steinernema feltiae* (Filipjev, 1934), and have shorter survival (respectively 6 and 4 months; Grewal, 2000).

With respect to IJ survival in relation to symbiont load, this has also been shown to be variable among species. For example, *S. carpocapsae* IJs exhibit a greater longevity in absence of their symbiont (Mitani et al., 2004) when compared to *S. scapterisci* (Emelianoff et al., 2007). Interestingly, the symbiont load in these two species is different, with 0.06 cfu/IJ for *S. scapterisci* vs. 43.6 cfu/IJ for *S. carpocapsae* (Sicard et al., 2003). Furthermore, studies have demonstrated that symbiont load in *S. carpocapsae* can be highly variable (ranging from 2.6 to 260 cfu/IJ) and that IJs with the higher *Xenorhabdus* load have higher mortality (Emelianoff et al., 2008). In addition, a trade-off between death rate of the nematode and their parasitic success has been observed (Emelianoff et al., 2008). While, aposymbiotic IJs survive longer, they are less efficient to succeed in infecting insects (Emelianoff et al., 2008). Nonetheless, the mechanisms underlying the impact on symbiotic load remain largely unknown.

To investigate the potential metabolic costs and gains of *Steinernema* IJs, we performed a comparative RNA-seq analysis of IJ stages in two symbiotic pairs: *Steinernema carpocapsae*-*Xenorhabdus nematophila* and *Steinernema puntauvense*-*Xenorhabdus bovienii* relative to their rearing conditions *in vivo* and *in vitro* (i.e., the role of the insect host) and in relation to the presence/absence of their symbiotic partners. Indeed, when the IJ stages emerge from the insect host, they are associated with their *Xenorhabdus* symbiont, so in order to study aposymbiotic IJ stages nematodes are obtained from *in vitro* reared eggs which aresymbiont-free (McMullen and Patricia Stock, 2014).

## Materials and Methods

### Bacterial Culturing and Nematode Rearing Conditions

Two *Steinernema-Xenorhabdus* pairs were used in this study, *Steinernema carpocapsae* (strain All) and *Xenorhabdus nematophila* and *Steinernema puntauvense* (strain Li6) and *Xenorhabdus bovienii*.

For each association, three different conditions were studied: (1) *in vivo*, nematodes were reared in *Galleria mellonella* larvae, (2) *in vitro*, colonized nematodes reared with their native symbiont and without an insect host, and (3) *in vitro* aposymbiotic, nematodes depleted of their symbionts and reared without an insect host. We choose these two associations because the *in vitro* rearing procedures of these two *Steinernema* species is routinely performed in the Stock laboratory and reliably produce aposymbiotic IJ progeny (McMullen and Patricia Stock, 2014; McMullen et al., 2017). *In vivo* rearing followed procedures described by Kaya and Stock (1997) with minor modifications (Kaya and Stock, 1997). Briefly, an inoculum of 100 IJs was used to infect one single last instar *G. mellonella* larva in a 1.5-ml microcentrifuge tube containing a piece of filter paper (Whatman grade 1). A total of 15–25 replicates were processed depending on the species tested and mortality rate. Insects were incubated at 25°C for 3–4 days, and upon death, cadavers were transferred to individual modified White traps (Kaya and Stock, 1997) to collect emerging IJs. Concurrently, for the *in vitro* conditions, insect cadavers were dissected to isolated 150 gravid females. The females were broken up to isolate eggs (not associated with *Xenorhabdus* symbiont) as described previously (Xu and Hurlbert, 1990; Kaya and Stock, 1997; Stock and Goodrich-Blair, 2012). *In vitro* rearing of the obtained eggs was done in liver-kidney agar supplemented with 0.1% sodium pyruvate following procedures described by Stock and Goodrich-Blair (2012). For *in vitro* colonized condition, eggs were inoculated onto bacterial lawns of their native symbionts. Plates were incubated in the dark at 25°C until IJs began to crawl up the side of the plate. Then, plates were transferred to a modified White trap setup (McMullen and Patricia Stock, 2014). IJs from all conditions were collected 4–5 days post emergence into the water.

### RNA Extraction and RNA-seq

A total of 10,000 IJs from each of the tested species were harvested from each of the rearing condition during the first 4–5 days of emergence. IJs were twice washed in sterile water, centrifuged at 22,000 *g* for 5 min, re-suspended in RNA protective storage solution (25 mM sodium citrate, 10 mM EDTA, and 0.7 g/ml ammonium sulfate, pH 5.2), snap frozen is liquid nitrogen, and stored at −80°C until RNA extraction. Sample preparation and sequencing for transcriptome analysis was done at the University of Arizona Genetics Core. The nematodes were ground with an Omni tip and rotor-stator running at >18 k rpm (>30 s) to disrupt their cuticle and facilitate RNA extraction. After grinding, debris was pelleted at high speed and the supernatant was then used in a Qiagen RNeasy Mini kit according to the manufacturer’s protocol (including the optional on-column DNase digestion step). Quality was assessed with RNA high sensitivity analysis kit (Advanced Analytical Technologies) and quantified with RiboGreen RNA assay kit (Quant-iT). RNA were clean-up and concentrate using column kit (Zymo). cDNA pool libraries were built for each condition with Illumina TruSeq RNA kit with average insert size of 196 bp for 2×100 bp paired-end sequencing. Library quality was assessed with next generation sequencing high sensitivity analysis kit (Advanced Analytical Technologies) and quantified with Illumina universal adapter-specific qPCR kit (Kapa Biosystems). All six samples were multiplex sequenced on a single lane of Illumina HiSeq 2500 with a second technical replicate lane.

### Transcriptomic Analysis

For each condition replicate, reads were trimmed and filtered using Trim Galore (version 0.6.4) and quality was assessed using FastQC (v0.11.9).^1^,^2^ The data were deposited on NCBI database: BioProject PRJNA766056; Biosample SAMN21601377, SAMN21601527, SAMN21601552 SAMN21604292, SAMN21604906, SAMN21604907; SRA SRR16057604 to SRR16057609. All the reads belonging to the same association were combined to assemble a *de novo* transcriptome assembly using Trinity (v2.10; Grabherr et al., 2011; Haas et al., 2013). In order to eliminate transcript reads from *Xenorhabdus*, a selection of transcripts were initially identified based on similarity with a custom protein database from the nematodes (*Steinernema carpocapsae*; *Steinernema scapterisci*; *Steinernema monticolum*; *Steinernema feltiae*; *Steinernema glaseri*; *Heterorhabditis bacteriophora*; *Strongyloides ratti*; *Loa loa*; *Dracunculus medinensis*; *Necator americanus*; Supplementary Table S1) using xblast. A *de novo* transcriptome assembly was produced for the tested species, *Steinernema carpocapsae* and *Steinernema puntauvense* (respectively associated with Transcriptome shotgun assembly (TSA) accession number GJLD00000000 and GJLE00000000 at NCBI). Open reading frames (ORFs) were identified in each transcriptome assembly, assessing for peptide sequence lengths >100 amino acids, using TransDecoder.^3^ Subsequently, reads from each condition were mapped back to the transcriptome assembly for the corresponding species, using Tophat (v2.1.1). The abundances of the transcripts and the differential expression were tested using two different methods: using Cufflinks (v2.2.1; Trapnell et al., 2012) and using edgeR (v 3.32.1; parameters FDR ≤ 0.01 and value of *p* ≤0.01; Robinson et al., 2009). Only contigs with >25 mapped reads across samples were considered for analysis. The gene expression tables were deposited as Gene Expression Omnibus (GEO) series under the accession number GSE185177. Orthology analysis of the differentially expressed transcripts between *in vivo* condition and both *in vitro* condition (colonized and aposymbiotic) for the two species of *Steinernema* were performed using Orthofinder (version 2.2.0; Robinson et al., 2009).

### Annotation, Analysis of Metabolism Pathway, and Enrichment Analysis

Annotation of the produced *de novo* transcriptome assemblies was produced using Augustus (version 3.3.3), considering training set based on *Caenorhabditis elegans* data (Stanke et al., 2006). GhostKOALA (Kanehisa et al., 2016) was used to associate proteins with KEGG orthology (KO) identifier and reconstruct KEGG metabolism pathway. Selection of transcripts and their expression were filtered by metabolic pathways using a custom shell script. Heatmaps of expression of transcripts were produced using the pheatmap package in R environment (values centered and scaled in the row direction; Kolde, 2019). The two technical replicates (two lanes of sequencing) were showed in the heatmaps for each condition. Annotation of the selected transcripts was confirmed by homology searches between the protein sequences and hidden Markov Models (HMM) profile using hmmscan in the HMMER web server (Potter et al., 2018).

Enriched functional terms of transcripts downregulated or upregulated in the two *in vitro*, conditions (colonized and aposymbiotic) were generated and sorted using the gene-list enrichment module of KEGG Orthology Based Annotation System intelligent (KOBAS-i version; Bu et al., 2021). KOBAS-i uses machine learning-based approach integrating seven functional class scoring (FCS) method and two pathway topology (PT) method. KOBAS-i associated the KEGG pathways and Gene Ontology (GO) terms with our selection of transcripts. The *C. elegans* KEGG pathway database was used for gene-list enrichment analysis. In addition, to establish the KEGG pathway and GO terms for each transcript, this method evaluated the enrichment of the transcripts. The enrichment analysis was performed using Fisher’s exact test with cut-off *p* < 0.05. Enriched terms associated with KEGG pathway were summarized in barplot representing the enrich ratio calculated as “input protein number”/ “background reference protein number” associated with enriched function.

### Comparison With Published Secretome of *Steinernema carpocapsae*

Excreted/secreted proteins (ESPs) release by *S. carpocapsae* IJs had been previously analyzed by Lu et al. (2017) who identified “venom protein” due to its exhibited toxicity. This study also identified a total of 472 ESPs using mass spectrometry. We compared the sequences of these ESPs with our transcript sequences by blastn similarity analysis and filtered sequences with at least 98% identity. We filtered the transcriptomic data for the potential ESPs for *S. carpocapsae* and produced heatmap of the expression of these genes (as described above). We also identified potential class of proteins involved and produced heatmap for both nematodes species based on the annotation of the transcripts.

### Validation by RT-qPCR

For gene expression validation, aliquots of 10,000 flash frozen IJ were homogenized manually in 1 ml of Trizol using an autoclaved micropestle. The Trizol manufacturer’s protocol was followed. cDNA was synthesized using the Bioline SensiFast cDNA Synthesis kit following manufacturer’s protocols. Quantitative real-time polymerase chain reaction (qRT-PCR) was used to measure gene expression across rearing conditions and species. Each primer set condition was optimized using Bioline SensiFast No ROX Sybr Master Mix and is summarized in the Supplementary Table S2.

Primers were designed against a variety of contigs for both species transcriptome assemblies using AmplifX (v2.0.7; Nicolas Jullien).^4^ A total of 32 pairs primers were used in this study to validate gene expression in *S. carpocapsae* and *S. puntauvense* for all three rearing conditions (Supplementary Table S2). Expression of housekeeping genes must be evaluated to calculate the relative expression of the genes of interest. Transcripts presenting no differential expression between the three conditions [*in vivo*, *in vitro* (colonized), and *in vitro* (aposymbiotic)] were selected. At least 100 reads across samples and common for the two studied nematode species were mapped, of which four were selected to be tested: actin (pfam00022), α-tubulin (cd02186), Minichromosome Maintenance proteins (MCM; pfam14551) and SYF2 splicing factor (pfam08231). Housekeeping genes associated with the best stability among the samples was established using NormFinder (Potter et al., 2018). Observed the best stability for Actin and Minichromosome Maintenance proteins (MCM) genes was 0.13 and 0.12, respectively, for *S. carpocapsae* and 0.12 and 0.18, respectively for *S. puntauvense*. By comparison, the α-tubulin gene exhibited a stability of 0.28 for *S. carpocapsae* and 0.21 for *S. puntauvense*, whereas statbility for SYF2 splicing factor gene present a stability of 0.28 for *S. carpocapsae* and 0.24 for *S. puntauvense*. We normalized the relative expression using the actin and MCM as housekeeping genes for both species. Primer efficiencies was determined for each transcript by running a standard curve and converted primers efficiency (E) was calculated as follows: [primer efficiency (%)/100] + 1. For each species, the two best housekeeping genes were used to determine the relative gene expression levels. We used a modified Pfaffl model to take into account multiple reference genes: $\begin{array}{l} \frac{(E_{GOI)}{}^{△CtGOI}}{\mathrm{GeoMean[(}E_{Ref)}{}^{△CtRef}]} \end{array}$ (Vandesompele et al., 2002; Hellemans et al., 2008). The condition of rearing *in vivo* was used as a sample calibrator to determine the delta Ct (∆Ct). The relative gene expression values were transformed by a logarithmic base 10 function to be plot. Differential expression was tested on the relative gene expression values (not transformed). We tested the normality of the data using the Shapiro–Wilk normality test in R environment, as well as the homogeneity of Variance using Levene’s test and the independency of the condition using Chi-squared test. If these three assumptions are validated (data normally distributed with common variance and independent group), we analyzed the variance using one way ANOVA associated with post-hoc Tukey HSD (Ranganathan, 2013) to compare the three conditions. Otherwise, we analyzed the variance with the non-parametric Kruskal-wallis rank sum associated with multiple pairwise comparisons using the dunn’s test in the R environment (dunn.test package; Dunn, 1964).

### Glycogen Extraction and Quantification

Glycogen was extracted from 10,000 IJ flash-frozen aliquots, that were homogenized manually in 25 mM citrate buffer (pH 4.2) and 2.5 g/L NaF on ice using an autoclaved micro pestle. Samples were then centrifuged at 14,000 × *g* for 10 min at 4°C to remove insoluble material. The upper phase was collected and transferred to a new tube. Then 10 μl of the resulting lysate is combined 90 μl “working reaction mix” prepared based on the EnzyChrom™ Glycogen Assay Kit (BioAssay Systems) protocol. To exclude glucose level background, sample blank was performed using a “working reaction mix” without enzyme A. For both nematode species and for rearing condition, we considered as a colorimetric assay (OD_570nm_) and standards served as a reference and following the manufacturer’s protocol. These procedures were repeated for two biological replicates for each rearing condition for *S. carpocapsae* and three biological replicates from *S. puntauvense*. Statistical analysis was applicable only for *S. puntauvense* (not enough replicates for *S. carpocapsae*). The distribution of the data was not normal so we analyzed the variance with the non-parametric Kruskal-wallis rank sum associated with multiple pairwise comparisons using the Dunn’s test in the R environment (dunn.test package; Dunn, 1964).

## Results

### Transcriptome Assemblies, Differential Expression, and Orthogroups

Between 1,370 and 1,470 million base pairs (bp) were generated for each sequencing run for each tested rearing condition. Removal of low-quality regions and adaptors yielded 13–14 million reads per condition. From these, we were able to produce two draft transcriptome assemblies of *Steinernema* for the present study (Table 1). Transcriptomic analyses of the *Xenorhabdus* are not showed in the current study due to the low number of sequenced reads belonging to the endosymbiont (more detail in Supplementary Table S3). For *S. carpocapsae*, a 51,538-contig draft transcriptome assembly was obtained with total length of 98,199,115 bp (N50 = 3,154), after transcript filtering and identification of open reading frames. For *S. puntauvense*, we obtained a 59,807-contig draft transcriptome assembly with a total length of 81,513,663 bp (N50 = 2,370) after transcript filtering and open reading frame identification.

**Table 1:** Statistical summary of transcriptome assemblies and number of differentially expressed transcripts in *in vitro* (colonized and aposymbiotic) and *in vivo Steinernema carpocapsae* and *Steinernema puntauvense* IJs.

Species	*S. carpocapsae*	*S. puntauvense*
*Transcriptome assembly statistics*		
Number of contigs	51,536	59,807
Total length (bp)	98,199,115	81,513,663
N50 (bp)	2,370	3,154
L50	9,376	9,913
GC (%)	50.38	49.73
*Number of differentially expressed transcripts*		
*In vivo* vs. *in vitro* aposymbiotic	3,332	2,844
*In vivo* vs. *in vitro* aposymbiotic: upregulated (cufflinks)	1,420	1,416
*In vivo* vs. *in vitro* aposymbiotic: upregulated (edgeR)	1,456	1,070
*In vivo* vs. *in vitro* aposymbiotic: upregulated (cufflinks/edgeR/both)	1,504	1,455
*In vivo* vs. *in vitro* aposymbiotic: downregulated (cufflinks)	1,480	1,196
*In vivo* vs. *in vitro* aposymbiotic: downregulated (edgeR)	1,500	1,322
*In vivo* vs. *in vitro* aposymbiotic: downregulated (cufflinks/edgeR/both)	1,828	1,389
*In vivo* vs. *in vitro* colonized	1,802	2,311
*In vivo* vs. *in vitro* colonized: upregulated (cufflinks)	779	1,193
*In vivo* vs. *in vitro* colonized: upregulated (edgeR)	579	757
*In vivo* vs. *in vitro* colonized: upregulated (cufflinks/edgeR/both)	798	1,213
*In vivo* vs. *in vitro* colonized: downregulated (cufflinks)	981	1,020
*In vivo* vs. *in vitro* colonized: downregulated (edgeR)	344	1,022
*In vivo* vs. *in vitro* colonized: downregulated (cufflinks/edgeR/both)	1,004	1,098
*In vitro* colonized vs. aposymbiotic	3,549	2,107
*In vitro* colonized vs. aposymbiotic: upregulated (cufflinks)	1,606	989
*In vitro* colonized aposymbiotic: upregulated (edgeR)	1,583	816
*In vitro* colonized vs. aposymbiotic: upregulated (cufflinks/edgeR/both)	1,671	1,032
*In vitro* colonized vs. aposymbiotic: downregulated (cufflinks)	1,478	955
*In vitro* colonized vs. aposymbiotic: downregulated (edgeR)	1,661	953
*In vitro* colonized vs. aposymbiotic: downregulated (cufflinks/edgeR/both)	1,867	1,075

**Figure 1:**
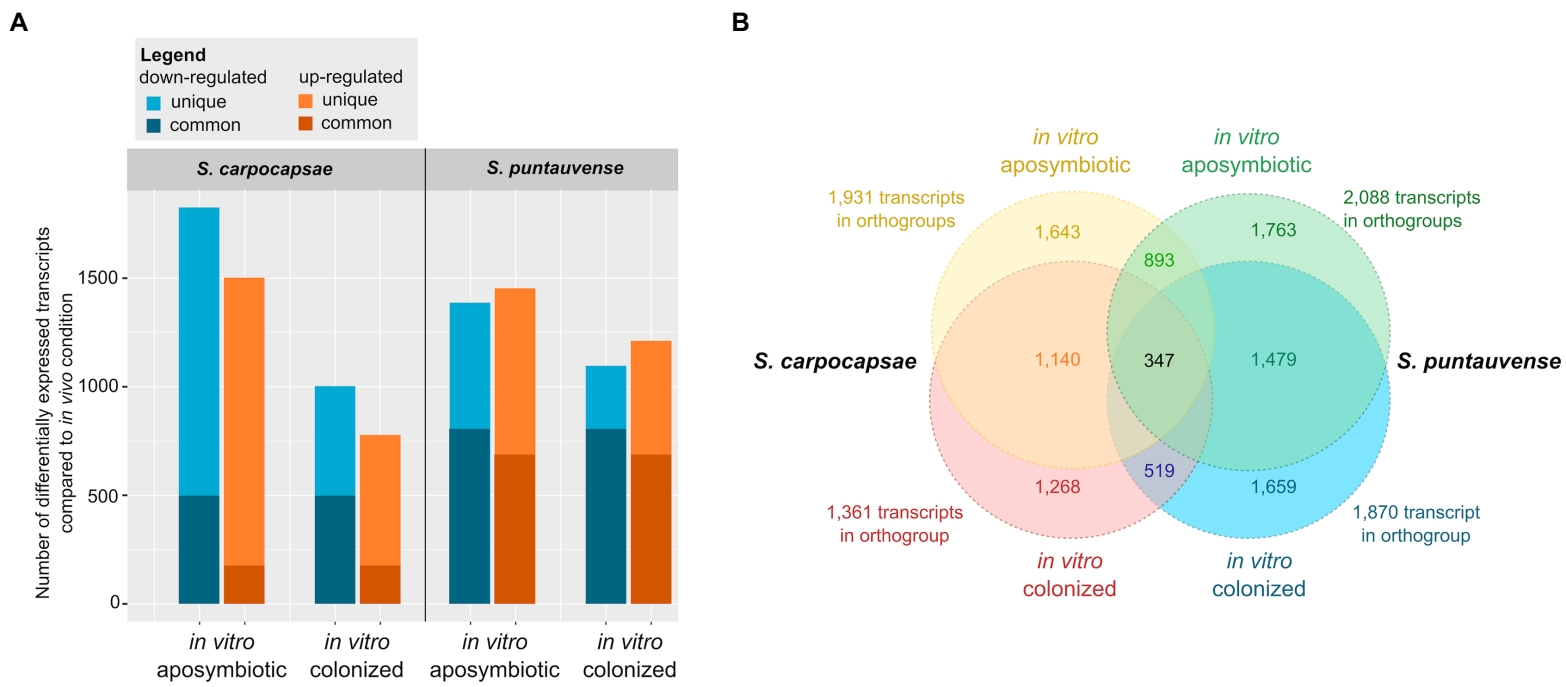
Summary of differentially expressed transcripts in *in vitro* reared (colonized and aposymbiotic) *Steinernema* IJs compared to *in vivo* reared *Steinernema* IJs. **(A)** Graph of transcripts number associated with significant downregulation or upregulation in the *in vitro* condition (colonized or aposymbiotic) were compared to the *in vivo* rearing condition. The differential expression was established using cufflink and/or edgeR. **(B)** Venn diagrams of orthogroups of differentially expressed transcripts shared among the *in vitro* colonized and *in vitro* aposymbiotic nematodes for the two *Steinernema* species. The orthogroups were identified with Orthofinder.

For *S. carpocapsae*, a total of 3,332 transcripts with differential expression between the *in vivo* vs. the *in vitro* aposymbiotic rearing condition were observed (significant differences using Cufflinks edgeR, or both analyses) while 1,802 transcripts with differential expression is observed between the *in vivo* vs. the *in vitro* colonized rearing condition (Table 1). In the *in vitro* aposymbiotic condition, a total of 1,480 transcripts (using Cufflinks) and 1,500 transcripts (using edgeR) were identified downregulated, while 1,420 transcripts (Cufflinks) and 1,456 transcripts (edgeR) were depicted as upregulated (Table 1; Figure 1A). In the *in vitro* colonized condition, a total of 981 transcripts (using Cufflinks) and 344 transcripts (using edgeR) were observed as downregulated, while 779 transcripts (Cufflinks) and 579 transcripts (edgeR) were identified as upregulated. Regarding the results between the *in intro* colonized vs. the *in vitro* aposymbiotic rearing condition, 3,549 transcripts exhibited differential expression (significant differences using Cufflinks edgeR, or both analyses): a total of 1,867 transcripts were observed downregulated while 1,671 were observed as upregulated (Table 1).

For S. *puntauvense*, 2,844 transcripts with differential expression were identified between the *in vivo* and the *in vitro* aposymbiotic rearing condition while 2,311 transcripts with differential expression is observed between the *in vivo* vs. the *in vitro* colonized rearing condition (Table 1). In the *in vitro* aposymbiotic condition, downregulated transcripts also varied depending on the tool used for the analysis, with 1,196 transcripts (Cufflinks) and 1,322 transcripts (edgeR) identified and upregulated transcripts also fluctuated between 1,416 (Cufflinks) and 1,070 transcripts (edgeR; Table 1; Figure 1A). In the *in vitro* colonized condition, a total of 1,020 transcripts (using Cufflinks) and 1,022 transcripts (using edgeR) were observed as downregulated, while 1,193 transcripts (Cufflinks) and 757 transcripts (edgeR) were identified as upregulated. Regarding the results between the *in intro* colonized vs. the *in vitro* aposymbiotic nematodes, 2,107 transcripts exhibited differential expression (significant differences using Cufflinks edgeR, or both analyses): a total of 1,075 transcripts were observed downregulated while 1,032 were observed as upregulated (Table 1).

Orthology analysis of the transcripts associated with differential expression in *in vitro* aposymbiotic condition revealed 893 orthogroups shared between *S. carpocapsae* and *S. puntauvense* were identified (Figure 1B). We performed a similar analysis for the transcripts associated with differential expression in *in vitro* colonized IJs and 519 orthogroups shared by the two species were identified. For both *Steinernema* spp., a more pronounced differential expression was observed in transcripts of *in vitro* aposymbiotic IJs than the *in vitro* colonized ones.

### Enrichment Analyses on Transcripts Associated With Differential Expression

**Figure 2:**
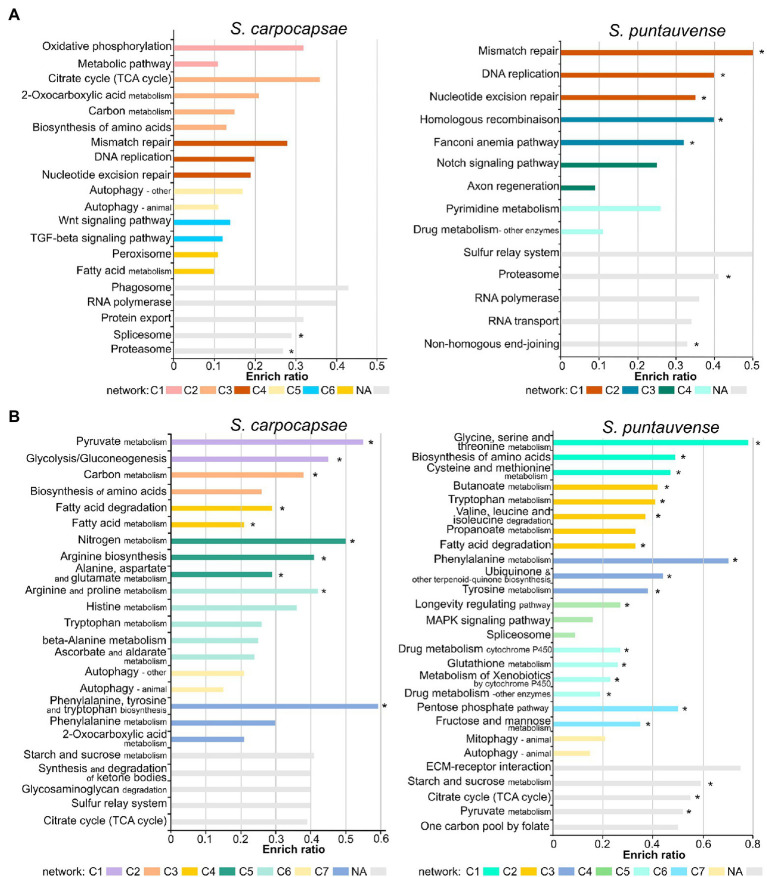
Barplot of enriched KEGG ratio of differentially expressed transcripts in *in vitro* aposymbiotic reared IJs when compared to *in vivo* reared IJs using i-KOBAS. The length of the bar represents the enrich ratio calculated as “input gene number”/ “backgound gene number.” The color of the bar represents network of protein representing modules identified by i-KOBAS based on *Caenorhabditis elegans* dataset. For networks where numerous modules are enriched only the top five are displayed. The “*” indicates enriched KEGG terms identified for both *in vitro* aposymbiotic and colonized reared IJs. **(A)** Enriched KEGG ration of upregulated transcripts. **(B)** Enriched KEGG ratio of downregulated transcripts.

Differentially expressed transcripts (based on edgeR and/or Cufflinks analyses) of *in vivo* and *in vitro* aposymbiotic IJs were analyzed for GO and KEGG pathway enrichment (Table 2; Figure 2). GO enrichment analysis of upregulated transcripts showed enriched GO terms in relation to cellular components and molecular function for both *Steinernema* species (Table 2). Specifically, enriched GO terms related to cellular anatomical entity (such as nucleus or cytoplasm) and those related to binding processes (including protein, nucleotide binding or ATP binding). GO enrichment analysis of downregulated transcript also showed several GO terms involved in cellular component and molecular function (Table 2). Conversely, the analysis of the downregulated transcript exhibited more enriched GO terms relative to catalytic activity (such as hydrolase, transferase, or oxidoreductase for *S. puntauvense*) are observed (Table 2).

**Table 2:** Top 10 enriched gene ontology (GO) terms of *in vivo* and *in vitro* aposymbiotic *S. carpocapsae* and *S. puntauvense* IJs.

Term	GO ID	Corrected *p*	GO ancestor
*Upregulated transcripts for S. carpocapsae*			
Nucleus	GO:0005634	2.69E-92	Cellular component
Cytoplasm	GO:0005737	1.63E-90	Cellular component
Protein binding	GO:0005515	6.55E-46	Molecular function; binding
Membrane	GO:0016020	2.18E-32	Cellular component
RNA binding	GO:0003723	1.90E-31	Molecular function; binding
Nucleotide binding	GO:0000166	2.28E-30	Molecular function; binding
Embryo development ending in birth or egg hatching	GO:0009792	2.55E-29	Biological process; developmental process
Nucleic acid binding	GO:0003676	3.69E-29	Molecular function; binding
Integral component of membrane	GO:0016021	3.04E-24	Cellular component
ATP binding	GO:0005524	9.39E-24	Molecular function; binding
*Upregulated transcripts for S. puntauvense*			
Nucleus	GO:0005634	1.22E-130	Cellular component
Cytoplasm	GO:0005737	1.54E-118	Cellular component
Nucleotide binding	GO:0000166	1.15E-79	Molecular function; binding
ATP binding	GO:0005524	1.84E-57	Molecular function; binding
Protein binding	GO:0005515	9.89E-53	Molecular function; binding
RNA binding	GO:0003723	3.79E-51	Molecular function; binding
Nucleic acid binding	GO:0003676	3.42E-46	Molecular function; binding
Nucleolus	GO:0005730	1.51E-33	Cellular component
Hydrolase activity	GO:0016787	1.54E-33	Molecular function; catalytic activity
Translation	GO:0006412	4.51E-29	Biological process; cellular process
*Downregulated transcripts for S. carpocapsae*			
Cytoplasm	GO:0005737	2.62E-126	Cellular component
Nucleotide binding	GO:0000166	3.75E-91	Molecular function; binding
ATP binding	GO:0005524	2.81E-81	Molecular function; binding
Metal ion binding	GO:0046872	2.63E-61	Molecular function; binding
Catalytic activity	GO:0003824	1.11E-60	Molecular function; catalytic activity
Cytosol	GO:0005829	9.35E-55	Cellular component
Transferase activity	GO:0016740	4.65E-54	Molecular function; catalytic activity
Hydrolase activity	GO:0016787	3.20E-52	Molecular function; catalytic activity
Nucleus	GO:0005634	3.26E-51	Cellular component
Mitochondrion	GO:0005739	1.03E-48	Cellular component
*Downregulated transcripts for S. puntauvense*			
Cytoplasm	GO:0005737	3.98E-92	Cellular component
Oxidation–reduction process	GO:0055114	2.07E-59	Molecular function; metabolic process
Catalytic activity	GO:0003824	2.45E-56	Molecular function; catalytic activity
Nucleotide binding	GO:0000166	6.33E-46	Molecular function; binding
Oxidoreductase activity	GO:0016491	1.35E-44	Molecular function; catalytic activity
Metal ion binding	GO:0046872	5.87E-44	Molecular function; binding
ATP binding	GO:0005524	3.55E-42	Molecular function; binding
Mitochondrion	GO:0005739	4.16E-39	Cellular component
Cytosol	GO:0005829	8.63E-34	Cellular component
Hydrolase activity	GO:0016787	1.86E-33	Molecular function; catalytic activity

An additional analysis was performed to identify enriched GO terms specific to the absence of *Xenorhabdus* symbionts in the *in vitro* aposymbiotic IJs by exclusion of the shared enriched GO terms (Supplementary Table S4). Results from this analysis showed variation between the two *Steinernema* species. In *S. carpocapsae*, upregulated transcripts revealed enriched GO terms involved in cellular process (such as proton transmembrane transport or cytoskeleton constituent) and biological regulation (such as regulation of translation); while in *S. puntauvense* they were involved in developmental process such as regulation of mesodermal cell fate specification and dauer exit (Supplementary Table S4). Similarly, downregulated transcripts in *S. carpocapsae* showed enriched GO terms involved in cellular process (such as cell–cell adhesion or tRNA aminoacylation). In *S. puntauvense*, depicted GO terms were those involved in developmental process (such as tissue development or animal organ morphogenesis) and metabolic process (such as trehalose metabolism or sphingolipid biosynthesis; Supplementary Table S4).

The KEGG pathway enrichment analysis of differentially expressed transcripts in the *in vitro* aposymbiotic condition revealed a strong variation between *S. carpocapsae* and *S. puntauvense* (Figure 2). However, similarities of upregulated transcripts for both species were related to replication and repair processes (such as DNA replication, RNA polymerase, and nucleotide excision repair), as well as in genetic information processing (such as proteasome and RNA polymerase; Figure 2A). Similarly, for both species, the enriched KEGG pathway of downregulated transcripts appeared related to carbohydrate metabolism (such as pyruvate, citrate, starch, and sucrose metabolism), lipid metabolism (such as fatty acid degradation), amino acid metabolism (such as tryptophan metabolism), and cellular processes (such as the autophagy pathway; Figure 2B).

A further analysis was performed to identify enriched KEGG pathway of differentially expressed transcripts in the *in vitro* colonized condition to compare with analysis of differentially expressed transcripts obtained from IJs reared *in vitro* and aposymbiotically (Supplementary Figure S1). Results were more similar among these two rearing conditions for *S. puntauvense* than for *S. carpocapsae*. For example, genes involved in the amino acid metabolism (such as histidine, tryptophan or arginine and proline metabolism) appear to be upregulated enriched KEGG pathways for the *in vitro* colonized condition while they were downregulated enriched KEGG pathways for *in vitro* aposymbiotic condition.

We further assessed the enriched KEGG pathway by category and our results support a general pattern of downregulation of numerous metabolic pathways in the *in vitro* aposymbiotic reared nematodes. Downregulated enriched KEGG pathways belonging to metabolism processes represented 50.82% of the entry for *S. carpocapsae* and even 61.88% for *S. puntauvense* (Supplementary Figure S2). The most downregulated enriched KEGG pathway appeared to be the carbohydrate metabolism (in particular, the starch and sucrose, glycolysis/gluconeogenesis, or pyruvate metabolism) and amino acid metabolism (in particular, arginine metabolism; Supplementary Table S4; Supplementary Figure S2). A general pattern of upregulation of numerous genetic information related function processing was observed in *in vitro* aposymbiotic IJs, transcripts relative to the RNA transport, proteasome or replication, and repair process. Upregulated enriched KEGG pathway belonging to genetic information processing represented 42.21% of the entries for *S. carpocapsae* and 58.92% for *S. puntauvense* (Supplementary Figure S2). Interestingly, transcripts involved in signaling and in general cellular processes were characterized by both upregulation and downregulation in the *in vitro* aposymbiotic IJs (Supplementary Table S4; Supplementary Figure S2).

### Downregulation of the Starch and Sucrose Metabolism

**Figure 3:**
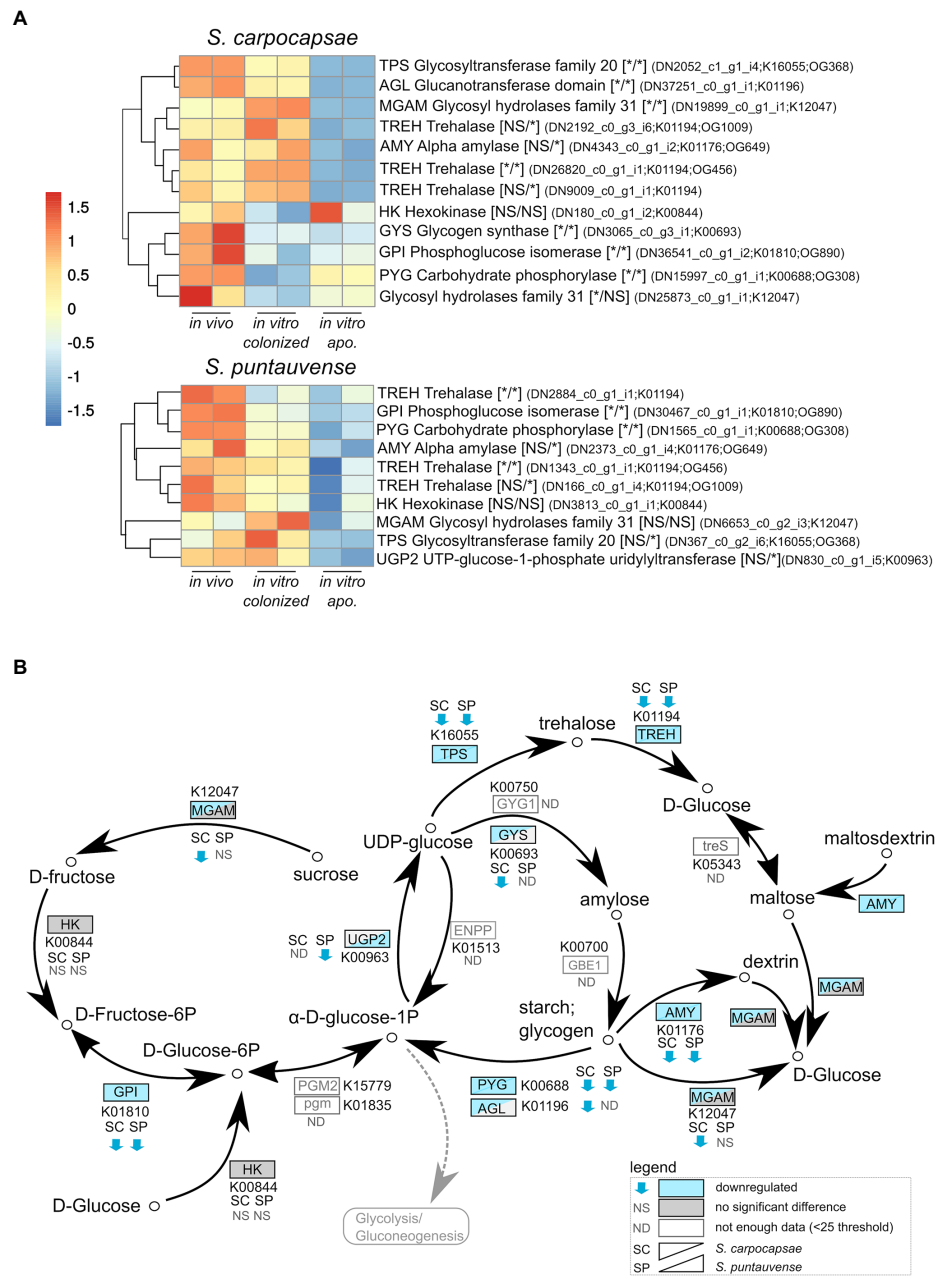
Transcriptomic analysis of genes involved in starch and sucrose metabolism. **(A)** heatmap of transcripts involved in the starch and sucrose metabolism for *Steinernema carpocapsae* and *Steinernema puntauvense* produced by pheatmap (values are centered and scaled in the row direction). For each condition, two technical replicates are showed in the heatmap. On the right side of the heatmap supplementary information is indicated including: annotation of the transcription (based on hmmer); significance of differentially expression based on cuffdiff and edgeR (* for significantly different and NS for not significant): first value for the *in vitro* colonized rearing condition and the second value for the *in vitro* aposymbiotic rearing condition; the name of the transcript; the KEGG pathway detected; when applicable the name of the orthogroup of the transcript shared between the two species (based on orthofinder analysis) is shown. **(B)** Map of the starch and sucrose metabolism pathway (ko00500), where the observed expression in the *in vitro* aposymbiotic reared IJs for both *Steinernema* species is shown.

Based on the KEGG enrichment results for carbohydrate metabolism, we further analyzed expression of transcripts involved in starch and sucrose metabolism (ko00500) for IJs reared *in vitro* (colonized and aposymbiotic; Figure 3). Specifically, transcripts involved in the glycogen pathway such as the carbohydrate phosphorylase *pyg* and the phosphoglucose isomerase *gpi* were down regulated in the colonized and aposymbiotic IJs for both tested *Steinernema* species (Figure 3A). Whereas the glycogen synthetase *gys* appears to be downregulated in *S. carpocapsae* and down-regulation of UTP-glucose-1-phosphate uridylyltransferase *ugp2* was only observed in *S. puntauvense* (Figure 3A). Unfortunately, not enough data were collected for these transcripts to statistically analyzed their expression for the other species (gene count was inferior to 25).

Downregulation was also observed for transcripts involved in the trehalose metabolism in both species, such as the trehalase *treh* amylase *amy* or glycosyltransferases *tps* (Figure 3A). Interestingly, this downregulation appears stronger for the *in vitro* aposymbiotic IJs than *in vitro* colonized IJs, two *treh* transcripts appear exclusively downregulated in absence of the symbiont for *S. carpocapsae*, same tendency was observed for one *tps* transcript and one *tre* transcript in the case of *S. puntauvense*. In summary, our results suggest a strong downregulation of glycogen metabolism for both *in vitro* reared IJs, while for trehalose metabolism genes, they appear to be more downregulated in the *in vitro* aposymbiotic nematodes.

**Figure 4:**
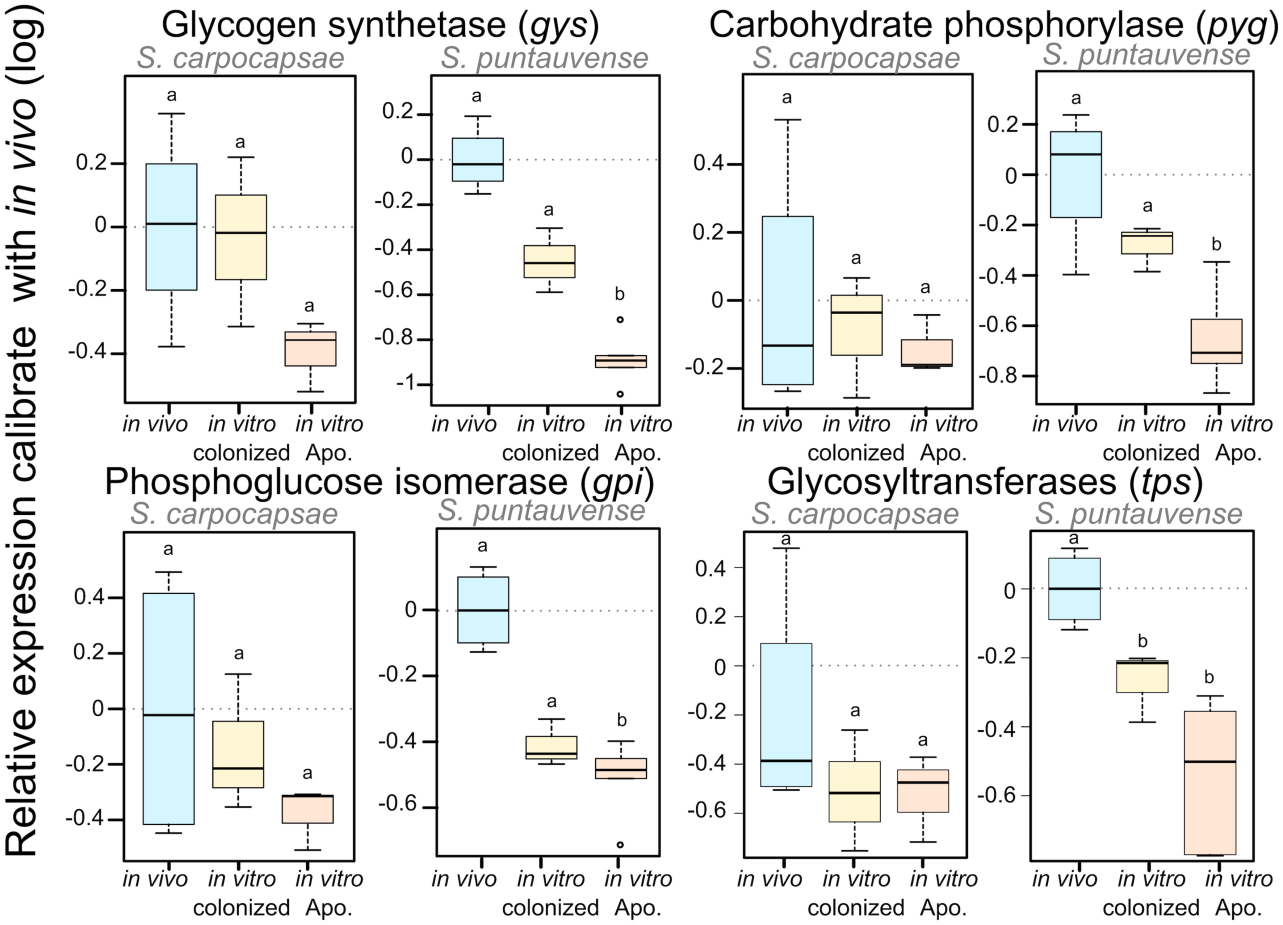
Boxplot of the relative expression of three transcripts involved in glycogen metabolism (*gys*, *pyg*, and *gpi*) using qRT-PCR. The relative expression was calculated using a modified Pfaffl model normalized with the two-housekeeping actin and MCM genes and calibrated with the value detected for the *in vivo* reared nematodes’ RNA. Boxplot shows the log of the calculated relative expression. Differential expression was tested by either one way ANOVA associated with *post-hoc* Tukey’s HSD or the non-parametric Kruskal-wallis rank sum associated with multiple pairwise comparisons using the Dunn’s test according to applicable condition. Bars labeled with the same letter are not significantly different from each other. Details of statistical test are shown in Supplementary Table S5.

We verified the downregulation of these transcripts by analysis of the relative expression using RT-qPCR of four genes: *gys* glycogen synthetase, *pyg* carbohydrate phosphorylase, *gpi* phosphoglucose isomerase, and *tps* glycosyltransferases. In *S. carpocaspae*, we observed a tendency for down-regulation of three of these genes (*gys*, *gpi*, and *tps*) at least for *in vitro* aposymbiotic IJs but it was not statistically significant (Figure 4; Supplementary Table S5). For *S. puntauvense*, *gys*, *pyg*, and *gpi* were significantly downregulated only in the *in vitro* aposymbiotic IJ condition (Figure 4). However, a tendency for downregulation of the three genes was also observed in the *in vitro* colonized IJs reared but this difference was not statistically significant (Figure 4; Supplementary Table S5). A significant difference was observed in the *in vitro* reared IJs (colonized and aposymbiotic) for the relative expression of the *tps* gene. Similarly, the observed downregulation of *tps* was stronger in the *in vitro* aposymbiotic IJ condition but this difference was not statistically significant (Supplementary Table S5).

**Figure 5:**
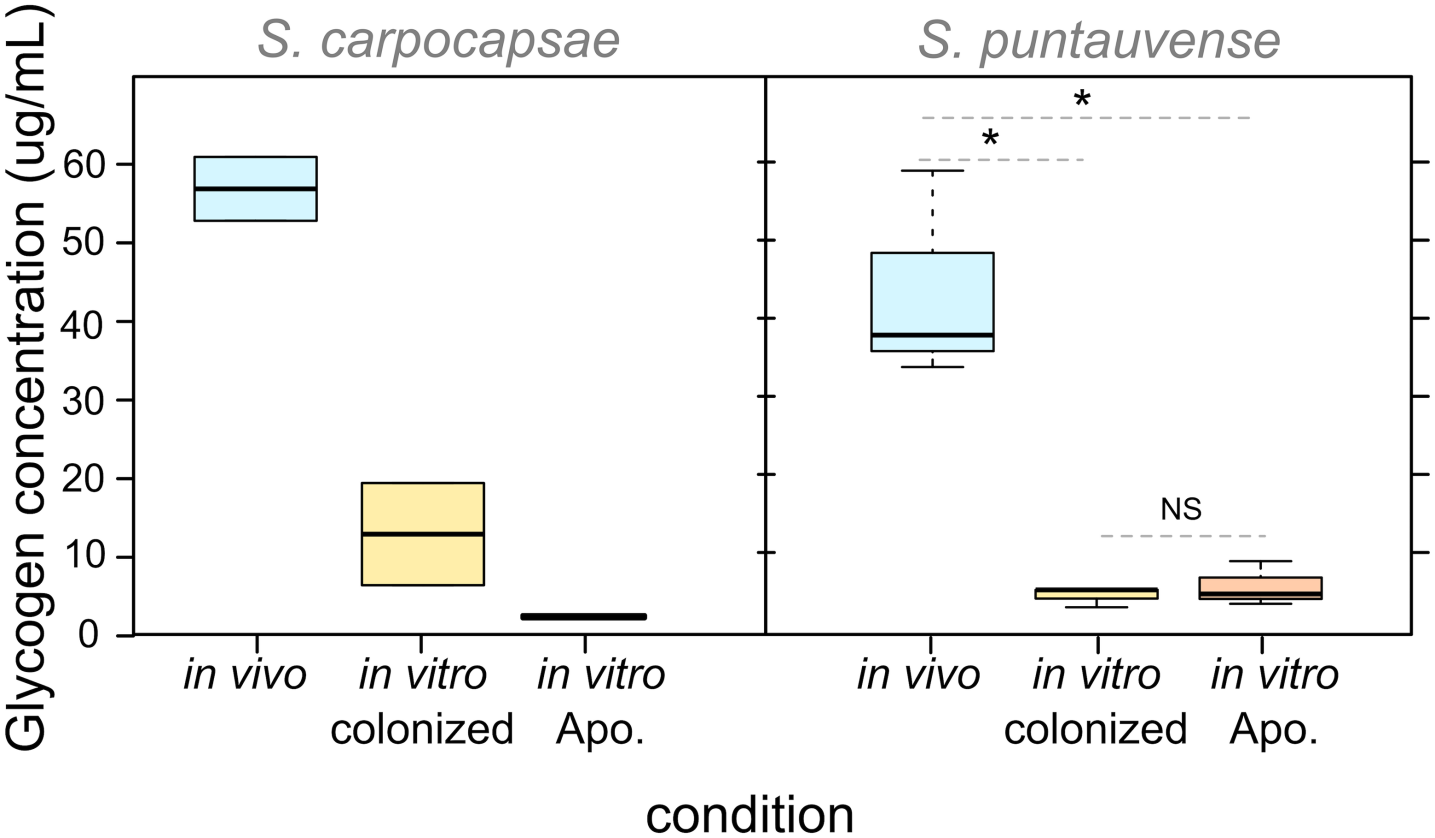
Boxplot of glycogen concentration detected in pool of 10,000 IJs reared in the three studied conditions (*in vivo*, *in vitro* colonized, and *in vitro* aposymbiotic). Their differential expression was tested by a non-parametric Kruskal-Wallis rank sum associated with multiple pairwise comparisons using the Dunn’s test (see detail in Supplementary Table S5). Asterix (*) indicates significant difference (value of *p* < 0.05), “NS” indicates no significant difference. Statistical tests were not performed on *S. carpocapsae* data due to the low number of replicates.

With respect to glycogen content, our observations showed that for both *Steinernema* species, IJs rear in *in vivo* present higher level of glycogen storage than nematodes reared *in vitro* under either condition (colonized or aposymbiotic; Figure 5). The difference appears to be significant for *S. puntauvense*; however, statistical analyses could not be performed on *S. carpocapsae* due to limit of available samples (Supplementary Table S5). Neither species displayed a difference in glycogen content for either *in vitro* condition, suggesting that the incidence of the symbiont has no effect on glycogen storage.

### Downregulation of Arginine Metabolism

**Figure 6:**
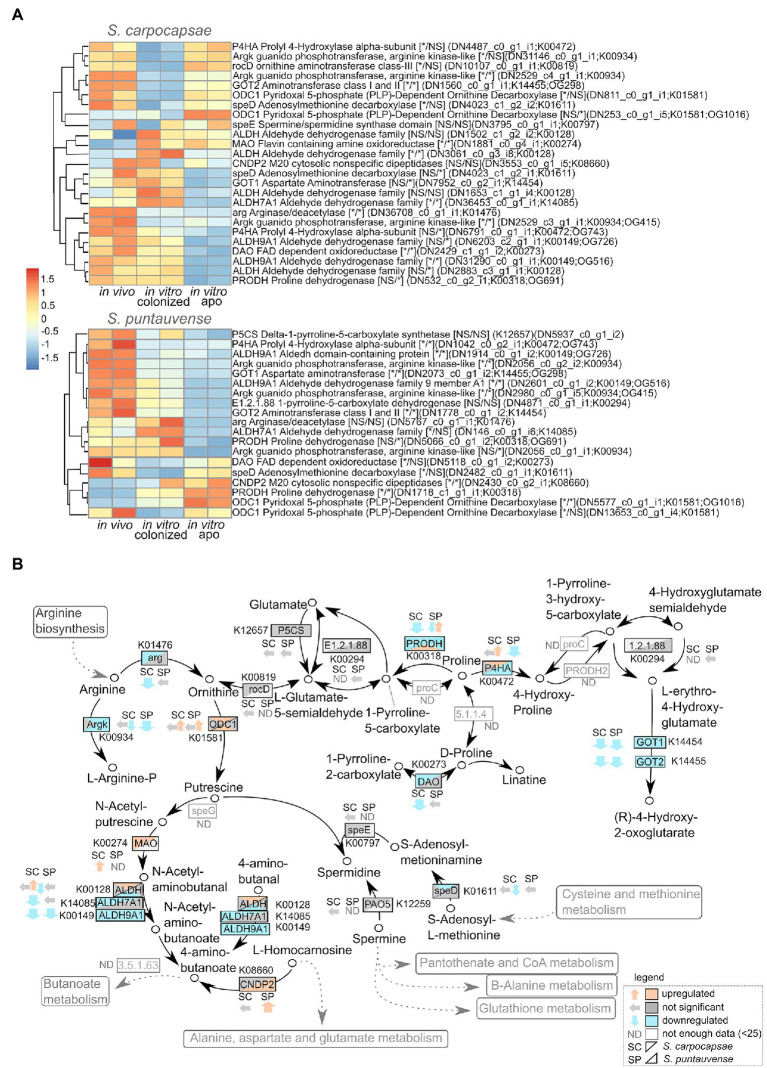
Transcriptomic analysis of genes involved in the arginine and proline metabolism. **(A)** Heatmap of transcripts involved in the arginine and proline metabolism for the species *S. carpocapsae* and *S. puntauvense* produced by pheatmap (values centered and scaled in the row direction). For each condition, two technical replicates are showed in the heatmap. On the right side of the heatmap supplementary information is indicated including: annotation of the transcription (based on hmmer); significance of differentially expression based on cuffdiff and edgeR, “*” denotes significant difference, NS for not significant; first value is for the *in vitro* colonized rearing condition and second value is for the *in vitro* aposymbiotic rearing condition; the name of the transcript; the KEGG pathway detected; if applicable the name of the orthogroup that belongs to the transcript shared between the two nematode species (based on orthofinder analysis). **(B)** Map of the starch and arginine and proline metabolism pathway (ko00330) where observed expression in the *in vitro* aposymbiotic reared nematodes for both *Steinernema* species is shown.

A detailed analysis on the expression of transcript involved in the arginine and proline metabolism (ko00330) was performed based on the KEGG enrichment results. Downregulation of numerous transcripts was observed for *in vitro* aposymbiotic condition for both of the tested *Steinernema* species (Figure 6). In particular, the transcripts involved in the arginine pathway, arginase/deacetylase *arg* (K01476) and arginase kinase *argk* (K00934) are downregulated for both species (a more significant difference was observed for *S. carpocaspae*; Figure 6A). Interestingly, our transcriptomic data suggest that there is a downregulation of arginine system transcripts in *in vitro* reared IJs (both colonized and aposymbiotic) in *S. carpocapsae*. However, in *S. puntauvense*, arginine genes are only downregulated in the *in vitro* aposymbiotic nematodes. This is also the case for transcripts encoding the proline dehydrogenase *prodh* (K00318), which seem to be downregulated mainly in aposymbiotic nematodes in both species.

**Figure 7:**
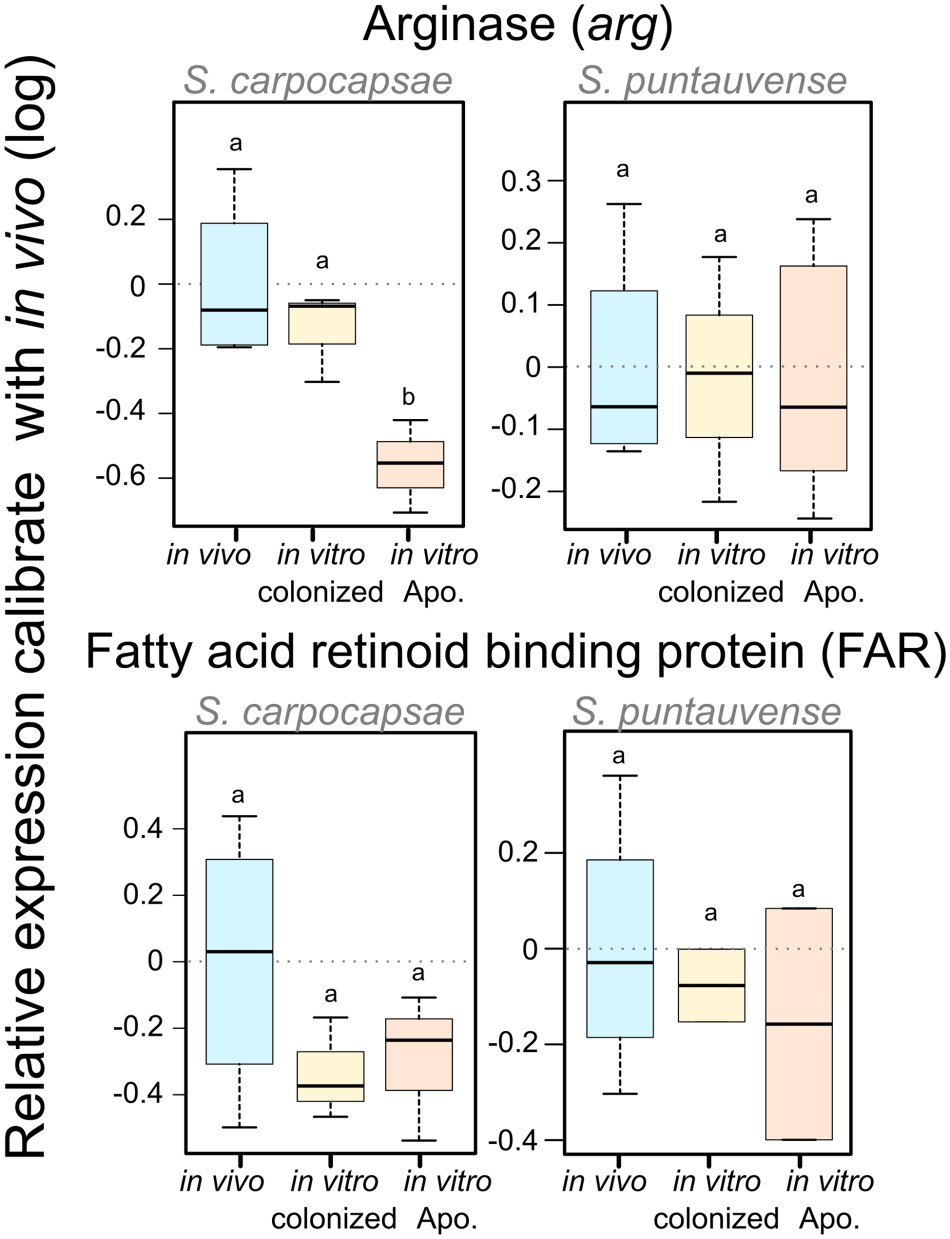
Boxplot of the relative expression of one transcript involved in trehalose metabolism (*tps*), one transcript involved in arginine metabolism (*arg*) and one transcript coding FAR protein using qRT-PCR. The relative expression was calculated using a modified Pfaffl model normalized with the two-housekeeping genes, actin and MCM that was calibrated with the value detected for the *in vivo* reared IJs’ RNA. Boxplot presents the log of the calculated relative expression. Differential expression was tested by either one way ANOVA associated with *post-hoc* Tukey’s HSD or the non-parametric Kruskal-Wallis rank sum associated with multiple pairwise comparisons using the Dunn’s test according to applicable condition. Bars labeled with the same letter are not significantly different from each other. See statistical test details in Supplementary Table S5.

We used the qRT-PCR to study the relative expression of the arginase gene (K01476). For *S. carpocapsae*, the relative gene expression of the arginase is significantly downregulated for the *in vitro* aposymbiotic IJs, while there was no significant differential expression in *S. puntauvense* (Figure 7; Supplementary Table S5).

### Regulation of the Longevity Metabolic Pathway

**Figure 8:**
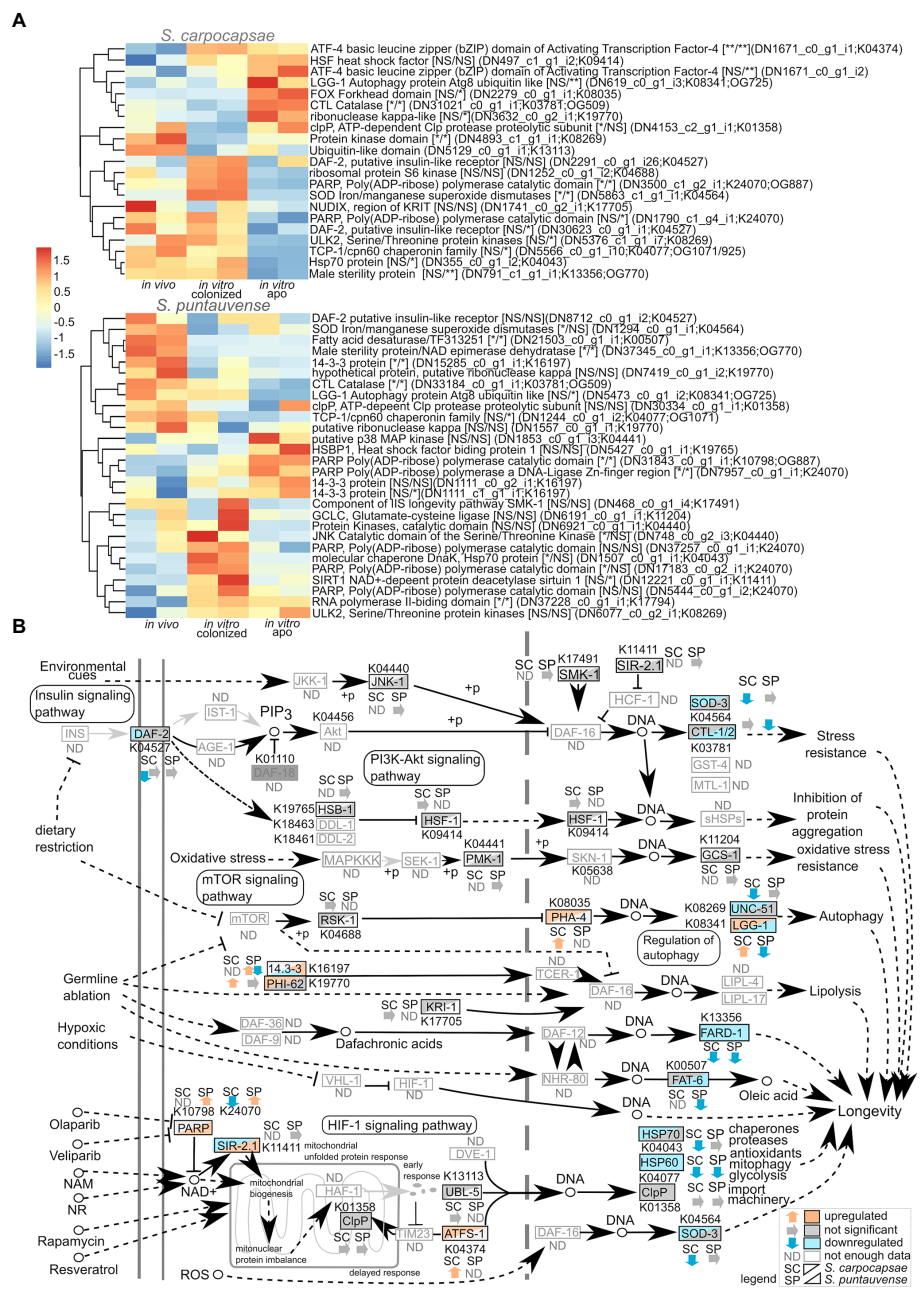
Transcriptomic analysis of genes involved in the longevity regulating pathway. **(A)** Heatmap of transcripts expression involved in the longevity regulating pathway for the species *S. carpocapsae* and *S. puntauvense* produced by pheatmap (values centered and scaled in the row direction). For each condition, the two technical replicates are showed in the heatmap. On the right side of the heatmap supplementary information is indicated including: annotation of the transcription (based on hmmer); significance of differential expression based on cuffdiff and edgeR (“*” denotes statistical significant difference, NS indicates no significant difference), first value for the *in vitro* colonized rearing condition and the second value for the *in vitro* aposymbiotic rearing condition; the name of the transcript; the KEGG pathway detected; if applicable, the name of the orthogroup that belongs to the transcript shared among the two species (based on orthofinder analysis). **(B)** Map of the longevity regulating pathway in nematodes (ko04212) where the observed expression in the *in vitro* aposymbiotic reared nematodes for *Steinernema* species is shown.

Based on the KEGG enrichment results suggesting differential expression of transcripts involved in the longevity metabolism pathway (ko04212), we examined the expression of transcripts involved in this pathway (Figure 8). First, we focused on genes involved in insulin/insulin-like signaling (IIS) pathway. The protein tyrosine kinase *daf-2* homolog of insulin-like growth factor 1 receptor (K04527) showed a tendency (not significant) of downregulation in the *in vitro* reared condition in *S. puntauvense* while a significant lower expression was observed in the *in vitro* aposymbiotic IJs in *S. carpocaspae*. Unfortunately, numerous genes involved in the IIS pathway such as the phosphatidylinositol 3-kinase *age-1* or the homolog of Forkhead box protein O *daf-16* exhibited low number of transcripts (below the established threshold of 25 gene count), thus we were unable to analyze their expression (Figure 8). Similarly, for genes involved in the mTOR signaling pathway, our analysis showed that there was a tendency for downregulation of the protein kinase *rsks/S6K* genes in *in vitro* aposymbiotic *S. carpocapsae* IJs, and an upregulation in the *in vitro* colonized condition.

We also focused on several stress resistance associated genes which changes in expression could indicate different mechanism influencing IJ longevity. Our results suggest downregulation of iron/manganese superoxide diamutases *sod-3* (K04564) of aposymbiotic (*in vitro* reared) *S. carpocapsae* IJs. With respect to *S. puntauvense*, a tendency for downregulation for *sod-3* was also observed in the *in vitro* aposymbiotic condition while significant downregulation was observed in the *in vitro* colonized IJs. With respect to catalase *ctl-1/2* (K03781), contrasting results were observed when comparing *in vitro* aposymbiotic IJs in both species, with an upregulation for *S. carpocapsae* and a down-regulation for *S. puntauvense*. A similar trend was depicted for genes involved in regulation of autophagy such as the autophagy protein *lgg-1*/*atg8* (K08341) or protein kinase *ulk2/unc-51* (K08269). Upregulation in the *in vitro* aposymbiotic condition was observed for *lgg-1* for *S. carpocapsae* whereas a downegulation was denoted for *S. puntauvense*. Gene *ulk2/unc-51* was down-regulated in *S. carpocapsae in vitro* aposymbiotic IJs while no significant difference was observed for *S. puntauvense in vitro* reared IJs (colonized and aposymbiotic). Downregulation of the chaperone proteins, heat shock protein *hsp70* and *hsp60* important to resistance of stress condition was observed in the *in vitro* reared aposymbiotic IJs for both species (only tendency was observed for *hsp70* in *S. puntauvense*).

**Figure 9:**
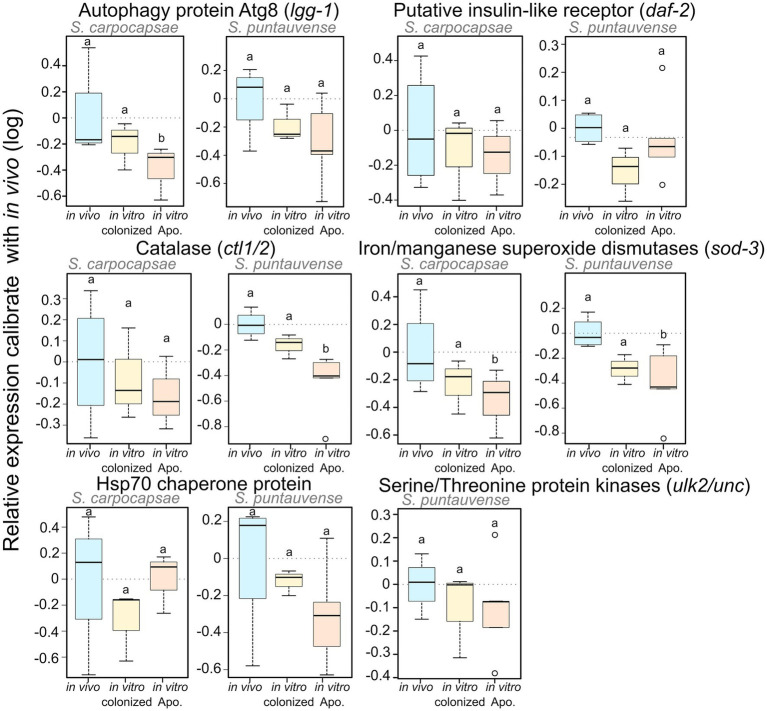
Boxplot of the relative expression of six transcripts involved in longevity regulating pathway (*lgg-1*, *daf-2*, *ctl1/2*, *sod-3*, *hsp70*, and *ulk2/unc*) using qRT-PCR. The relative expression was calculated using a modified Pfaffl model normalized with the two housekeeping genes, actin and MCM and calibrate with the value detected for the *in vivo* reared nematodes RNA. Boxplot present the log of the calculated relative expression. Differential expression was tested by either one way ANOVA associated with *post-hoc* Tukey’s HSD or the non-parametric Kruskal-Wallis rank sum associated with multiple pairwise comparisons using the Dunn’s test according to applicable condition. Bars labeled with the same letter are not significantly different from each other. See details of statistical test in Supplementary Table S5.

To test the expression of transcripts involved in the longevity metabolism pathway, we evaluated the relative expression by qRT-PCR of six genes: *sod-3*, *ctl-1/2*, *lgg-1*, *ulk2/unc-51*, *daf-2*, and *hps70*. We demonstrated a significant lower relative expression for: *lgg-1* in *S. carpocapsae in vitro* aposymbiotic IJs, *ctl1/2* in *S. puntauvense in vitro* aposymbiotic IJs and *sod-3* in the *in vitro* aposymbiotic IJs of both species (Figure 9; Supplementary Table S5). Only a tendency for downregulation was observed for at least the *in vitro* aposymbiotic condition for: *ctl-1/2* for *S. carpocapsae*, *lgg-1*, *ulk2/unc-51* and *hsp70* for *S. puntauvense* (optimization of qPCR for *ulk2*/*unc51* was not successful for *S. carpocapsae*; Figure 9). The qRT-PCR did not support contrasting results between the two *Steinernema* species for the expression *ctl1/2* or *lgg-1* as suggested by the transcriptomic analysis. However, the qRT-PCR was based on different RNA samples than those used for the transcriptomic analysis, and this may explain this may outcome. In addition, for *S. carpocapsae* the values obtained for *in vivo* reared IJs exhibited high variability which impacted further statistical analyses.

### Absence of the Symbiont Induces Change of Expression of the Venom Protein

**Figure 10:**
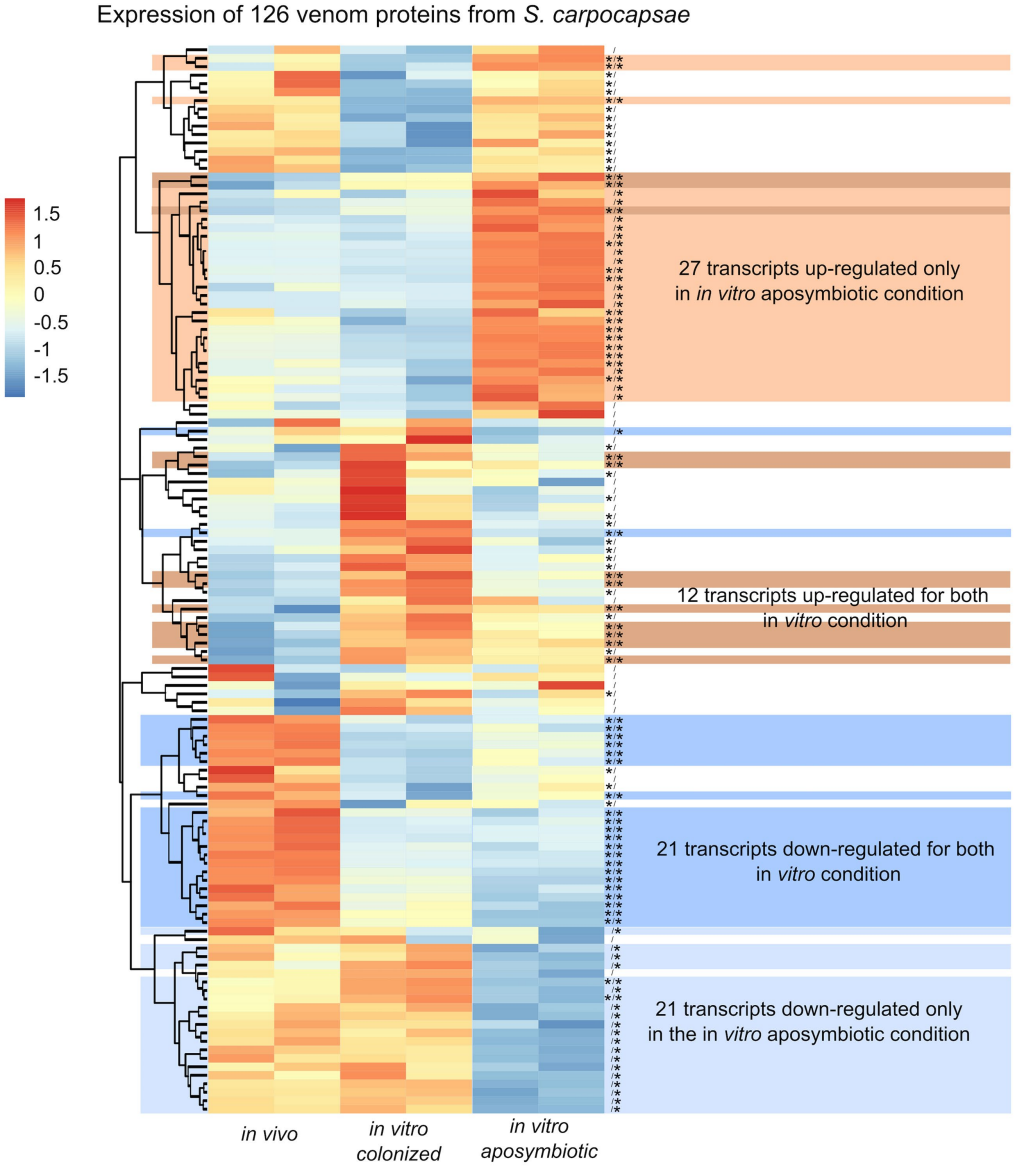
Heatmap of *S. carpocapsae* transcripts expression homologous to identified *S. carpocapsae* venom proteins by pheatmap. On the right side of the heatmap supplementary information are indicated: significance of differentially expression based on cuffdiff and edgeR (* for significantly different or bank for not significant), first value for the *in vitro* colonized rearing condition and the second value for the *in vitro* aposymbiotic rearing condition; group of transcript associated with differential expression only in the *in vitro* aposymbiotic reared nematodes or for both *in vitro* (colonized and aposymbiotic) reared nematodes.

Along with the analysis of the differential expressed genes, we observed classes of genes not directly associated with metabolic pathway but previously described in excreted/secreted (ES) products of *Steinernema* (Dillman et al., 2015; Lu et al., 2017). In total, 472 proteins, named as venom protein, were described in ES product of *S. carpocapsae* (Lu et al., 2017). We identified 126 transcripts homologous to venom proteins in the produced transcriptomic assembly of *S. carpocapsae* (Supplementary Table S6). Among these transcripts, we observed some proteins classes to be more abundant, including actin p (*n* = 8), ubiquitin (*n* = 10), trypsin serine protease or trypsin inhibitor (*n* = 7), serine carboxypeptiase (*n* = 4), and fatty acid and retinol-binding proteins (*n* = 5; Supplementary Table S6). Eighty-two of these transcripts exhibited significant differences in their expression when comparing colonized *in vivo* with aposymbiotic *in vitro* reared IJs. Similarly, 77 transcripts showed differential expression when contrasting colonized *in vivo* reared and colonized *in vitro* IJs (Figure 10). In total, 21 transcripts were down-regulated, and 27 transcripts were upregulated only in the *in vitro* aposymbiotic condition (Figure 10). Among the 27 upregulated genes, 14 were downregulated in the *in vitro* colonized nematodes. These results suggest that absence of *Xenorhabdus* symbionts may influence the secretion of venom proteins.

**Figure 11:**
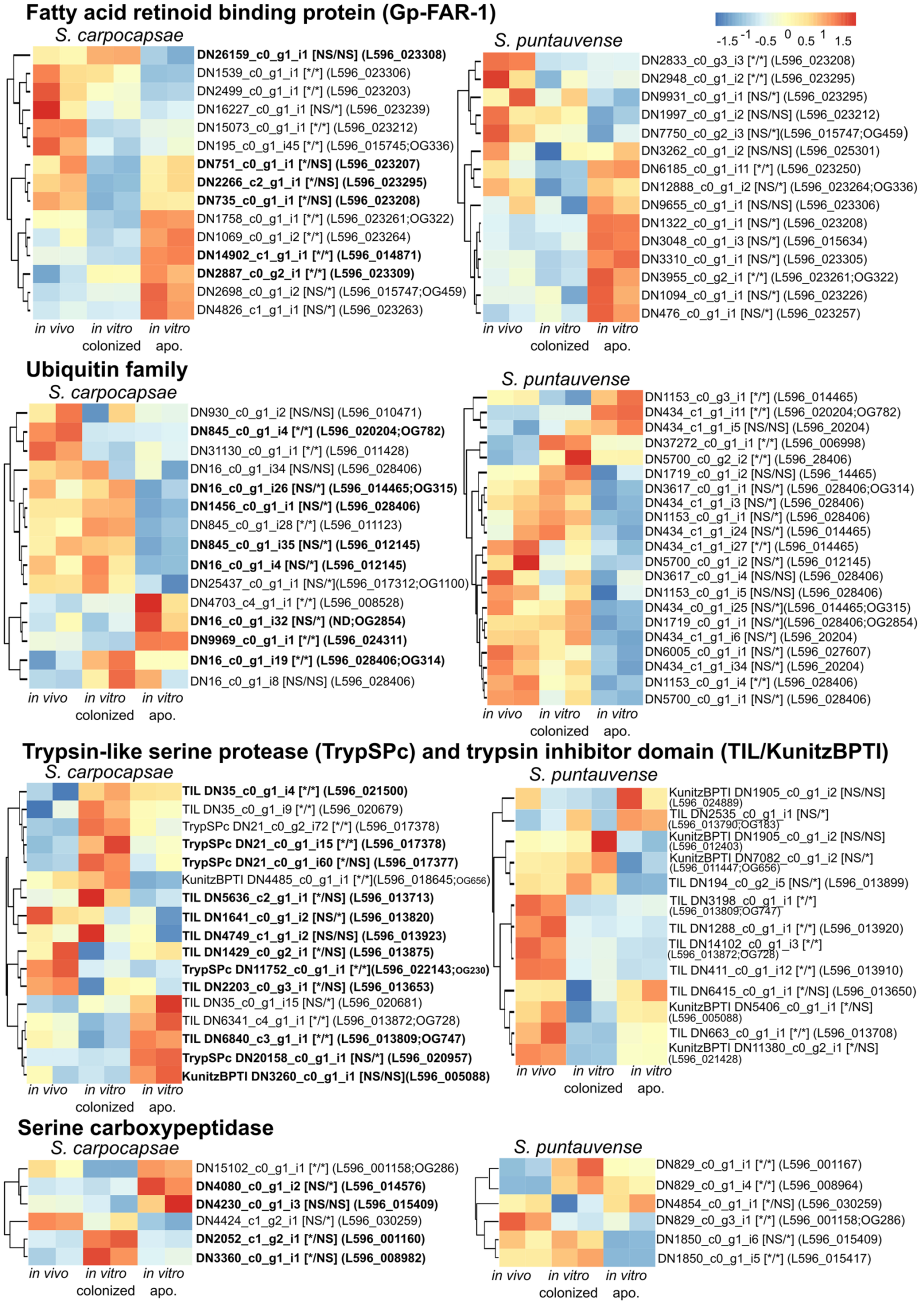
Heatmap of expression of transcripts identified as Fatty acid and retinoid binding protein, ubiquitin, trypsin-like serine protease, trypsin inhibitor domain and serine carboxypeptidase by pheatmap. On the right side of the heatmap supplementary information is indicated: the name of the transcript; significance of differentially expression based on cuffdiff and edgeR (“*” denotes statistically significant difference, NS indicates no significant difference), first value for the *in vitro* colonized rearing condition and the second value for the *in vitro* aposymbiotic rearing condition; homologous genes in *S. carpocapsae* genome; if applicable the name of the orthogroup of transcript shared among the two species (based on orthofinder analysis) is shown.

We further focused on a differentially expressed transcript that is specifically related to symbiont loss (Figure 11). First, we analyzed expression of 15 transcripts encoding fatty acid and retinol-binding (FAR) proteins in both nematode hosts. Most of these transcripts for both species exhibited a significant differential expression of transcripts in aposymbiotic IJs reared *in vitro* (Figure 11). For *S. carpocapsae*, six transcripts were upregulated, and the five others were downregulated, while for *S. puntauvense*, seven transcripts were upregulated and four were downregulated. Second, we analyzed the expression of transcripts encoding the ubiquitin that we observed in high number among the venom proteins. More ubiquitin protein appears to be downregulated (eight for *S. carpocapsae* and 13 for *S. puntauvense*) than upregulated in the *in vitro* aposymbiotic condition (four for *S. carpocapsae* and four for *S. puntauvense*). Most of this differential expression is specific to IJs that do not carry symbionts (Figure 11). With respect to the expression of trypsin-like serine protease (TrySPc) and trypsin inhibitor (TIL), we also observed a strong difference between the *in vitro* colonized and *in vitro* aposymbiotic nematodes for both *Steinernema* species. However, contrasting results were denoted among the two species, regarding the TIL protein, which showed a strong down-regulation in *S. puntauvense* (seven transcripts of eight) while only a tendency of upregulation was denoted in *S. carpocapsae* (five upregulated and two downregulated; Figure 11). Another protein indicated as a venom protein, the serine carboxypeptidase showed regulation specific to the presence or absence of symbiont. For example, in *S. carpocapsae*, the serine carboxypeptidase homologs to L596_001160 and L596_008982 were upregulated in the *in vitro* colonized nematodes while those homologs to L596_014576 and L596_001158 were upregulated only in the *in vitro* aposymbiotic nematodes. In addition, this regulation of serine carboxypeptidase might be species depend, and the transcript homologous to L596_001158 was up regulated for in aposymbiotic *S. carpocapsae* and downregulated in *S. puntauvense*. Other venom proteins, such as calcineurin-like phosphoesterase, zinc carboxypeptidase, ShK domain, and TILa domain also exhibited a strong regulation in aposymbiotic IJs for both *Steinernema* species (Supplementary Table S6). Although strong regulation was observed, there was no common pattern among these proteins.

We tested the expression of the transcript encoding fatty acid and retinol-binding (FAR) orthologous to L596_023261 gene from *S. carpocapsae* genome reference (AZBU02000000) results by a qRT-PCR assay (Dillman et al., 2015). The transcriptomic analysis showed this transcript upregulated in the *in vitro* aposymbiotic condition and downregulated in the *in vitro* colonized condition for both nematodes (Figure 11). However, the relative expression established by qRT-PCR did not show significant differential expression for the FAR gene in both nematode species (Figure 7).

## Discussion

### Absence of an Insect Host Affects Important Metabolic Pathways of *Steinernema* IJs

For more than 90 years, *Steinernema* entomopathogenic nematodes have been successfully used as biological control agents of a wide range of soil-inhabiting insect pests (Stock, 2005). Currently, several species of *Steinernema* are cultured and commercialized as biocontrol agents to kill insects (Shapiro-Ilan et al., 2016; Saleh et al., 2019). They have been demonstrated to be good biopesticides for the wide range of insects (Saleh et al., 2019). Both *in vivo* and *in vitro* methods have been developed for their mass-production and commercial development (Bedding, 1981; Shapiro-Ilan et al., 2016). However, most *Steinernema* intended for commercial application are produced *in vitro* due to the lower cost associated when compared with *in vivo* rearing (Saleh et al., 2019). The fitness of mass-production of entomopathogenic nematodes was previously addressed by Hatab and collaborators (Hatab et al., 1998). The authors concluded that lipid composition of IJs is influenced by the insect host and by the medium used for *in vitro* rearing. For example, in *S. glaseri*, a variation of lipid content was observed ranging from 50.7% to 60.4% depending on the culturing methods (Hatab et al., 1998). The authors also proposed that the lipid composition of *in vitro* media very different than the insect host might influences the lipid composition of the IJs (Hatab et al., 1998). In this study, we provide further evidence for this observation. Here, we observed the downregulation of the carbohydrate, amino acid, and lipid metabolic pathways in IJs that were reared *in vitro* when compared to IJs reared *in vivo*, both with and without the symbiont. These results, paired with the observation of upregulation of genes involved in genetic process such as DNA replication or mismatch repair provide further evidence that *in vitro* rearing causes a strong disturbance in several biological functions of *Steinernema* IJs.

Previous studies have reported that *in vitro* reared IJs have a lower bacterial load and have a shorter lifespan when compared to those reared *in vivo*. For example, Flores-Lara et al. (2007) reported that IJs reared in last instar larvae of *G. mellonella* exhibited higher bacterial load (expressed as CFU/IJs) than those reared *in vitro* (i.e., in lipid agar). Peterson et al. (2019) also demonstrated that the longevity of IJs reared in liver-kidney agar (LKA) was shorter (with lower survival rates at 8-week post emergence) when compared to those reared in an insect host. In this study, our transcriptomic data revealed a strong disturbance of several metabolic pathways of *in vitro* colonized and aposymbiotic IJs, suggesting a level of stress induced by this rearing method. We speculate these observations may have direct connotations when considering media for *in vitro* rearing and mass production of *Steinernema* nematodes. Further investigations on this subject are warranted.

### Longevity Pathway and Impact of Downregulated Glycogen, Trehalose, and Arginine Pathway

Although mechanisms of longevity are known to be variable among organisms, some conserved mechanisms have been ascertained (Kimura et al., 1997; Uno and Nishida, 2016). Historically, the free-living nematode *C. elegans* was established as a prominent model organism in aging research (Zečić and Braeckman, 2020). One of the first identified pathways involved in the regulation of the aging process was the insulin/insulin-like growth factor-1 signaling (IIS) pathway (Uno and Nishida, 2016). In the model *C. elegans*, three key components of the IIS pathway were determined: *daf-2*, homolog of the insulin/insulin-like growth factor-1 receptor; *age-1*, homolog of phosphatidylinositol 3-kinase; and *daf-16*, homolog of the forkhead box FoxO transcription factor (Uno and Nishida, 2016). Kimura et al. (1997) showed that *daf-2* appears to mediate the endocrine signaling, indeed a decrease of *daf-2* signaling impacts development changes (Kimura et al., 1997). More specifically, it has also been proposed that neuronal *daf-2* activity in *C. elegans* allows maintenance of low level of free radical-scavenging enzymes, such as *sod-3* and *ctl-1* by antagonizing the *daf-16* transcription factor and thus protecting neurons from oxidative damage (Wolkow et al., 2000). Recently, a transcriptomic analysis conducted by Xie et al. (2020) showed that when *S. carpocapsae* nematodes are under thermal stress, there is an upregulation of heat shock protein, *sod*, as well as *daf-16* and a downregulation of *daf-2*. In this respect, our transcriptomic data showed a down-regulation of *sod-3*, *ctl-1/2* and chaperone proteins in the *in vitro* rearing of aposymbiotic IJs, but not a significant difference for *daf-2*. Thus, our results suggest that absence of symbiont triggers an expression modification of similar genes that those observed by Xie et al. (2020) in their thermal stress study although the regulation appears opposite.

Numerous studies on *daf-2* mutants in *C. elegans* have shown a metabolic remodeling compared to wild-type strains (Zečić and Braeckman, 2020). For example, *daf-2* mutants exhibit upregulation of glycogen synthetase, as well as increased lipid and glycogen storage (Depuydt et al., 2014). It appears that it is not the glycogen alone or glucose level which shortens the lifespan of *C. elegans*, but their interference with the *daf-2* antiaging signaling (Gusarov et al., 2017). It was proposed that AMP-activated kinase (AMPK), a conserved cellular energy sensor, is glycogen-dependent, although the underlying mechanisms remain unclear (Gusarov et al., 2017; Gusarov and Nudler, 2018). In addition, the glycogen appears to have a protective role in *daf-2* mutants in conditions of anoxic and hyperosmotic environment (Frazier and Roth, 2009; Lamacchia et al., 2015).

In this study, we demonstrated a lower glycogen level in *in vitro* reared IJs, which may influence the longevity pathway and lifespan of the nematodes. Qiu and Bedding (2000) showed that 85%–90% of the dry weight of *S. carpocapsae* IJs is composed of lipids, glycogen, proteins, and trehalose. Glycogen plays an important role in the infectivity of *Steinernema* IJs (Patel et al., 1997; Wright et al., 1997), especially during the initial stages of the insect host infection (although variable among *Steinernema* species; Wright et al., 1997). Glycogen has also been demonstrated to be an alternative energy reserve in IJs (Qiu and Bedding, 2000).

Our results showed that *in vitro* reared IJs have a lower concentration of glycogen content when compared to those reared *in vivo* suggesting that the insect host likely provides key nutrients (e.g., glucose) for glycogen allocation. In this respect, it has also been shown that *Steinernema* IJs modify carbohydrate composition of the insect host. For example, when *Galleria mellonella* larvae were infected with *S. feltiae* and *S. affinis*, they exhibited lower level of glycogen but a higher level of trehalose (Zółtowska and Lopieńiska-Biernat, 2006). Interestingly, our data revealed that trehalose metabolism was more downregulated in aposymbiotic IJs when compared those that were colonized in the *in vitro* reared nematodes. Similarly, a previous study by Gal et al. (2001) showed that a shift from glycogen to trehalose synthesis is observed during dehydration stress (Gal et al., 2001). In this respect, Solomon et al. (Gal et al., 2001) observed a reduction of the expression of the glycogen synthase in *S. feltiae* IS-6 strain under desiccation stress (after 24 h dehydration). The authors concluded that desiccation tolerance in *S. feltiae*, may be correlate with elevated levels of trehalose (Gal et al., 2001).

Similarly, in *C. elegans*, it has been demonstrated that a reduction of the glycogen synthetase expression leads to a metabolic shift from glycogen to trehalose (Seo et al., 2018). Futhermore, Honda et al. (2010) showed that in *C. elegans*, RNAi inactivation of *tps* genes was associated with a shorter lifespan and a reduce tolerance to heat stress (Honda et al., 2010). High levels of trehalose upregulate transcription factor important for autophagy, such as *lgg-1*, *bec-1*, *sqst-1* and *unc-51* and affect the lifespan of the nematodes (Seo et al., 2018). In this study, a downregulation of the trehalose pathway was observed in aposymbiotic *S. carpocapsae* IJs reared *in vitro*, whereas an upregulation was denoted in colonized IJs reared *in vitro*. These results suggest that in absence of symbiont a potential metabolic shift in nutrient allocation from glycogen to trehalose content is not possible.

The TOR signaling pathway has also been identified as involved in aging regulation (Uno and Nishida, 2016). In *C. elegans*, inhibition of the TOR signaling increases the lifespan by regulation of mRNA translation through the *rsks-1*/S6 kinase (S6K; Lapierre and Hansen, 2012; McQuary et al., 2016). The *rsks-1*/S6 kinase (S6K) is involved in the regulation of numerous proteins known as factors of longevity in *C. elegans*, such as the transcription factor *pha-4* (homolog of FoxA forkhead box A) involved in autophagy regulation or the AMPK cellular energy sensor involved in adaptation to low-energy conditions (Lapierre and Hansen, 2012; Uno and Nishida, 2016). Interestingly, a proteomic study of *rsks-1*/S6 mutants highlighted over-expression of arginine kinase *argk-1* (McQuary et al., 2016). In this study, the authors showed that overexpression of *argk-1* can extend lifespan of *C. elegans* and that the arginine kinase *argk-1* is required for lifespan extension of *C. elegans* S6K deficient toward regulation of energy sensor AMPK (McQuary et al., 2016). More recently, Rozanov et al. (Rozanov et al., 2020) highlighted the importance of arginine kinase expression in the aging process in *C. elegans* but with different outcome. The authors demonstrated that transcription factor hlh-2 was involved in the regulation of the expression of arginine kinases acting in *C. elegans* pro-aging. The decrease of arginine kinase expression appears to generate an alteration in energy metabolism and ROS homeostasis and mediates health-beneficial effects (Rozanov et al., 2020). In the present study, we showed a tendency for down-regulation of genes involved in arginine metabolism (both *arg* arginase and *argk* arginine kinase) in IJs reared *in vitro*. Arginine kinase has been described as a significant component of the energy metabolism in *S. carpocapsae* IJs, suggesting it may play a key role in the aerobic/anaerobic metabolic transition (Platzer et al., 1999). This study also suggested that the presence of oxygen stimulates phosphoarginine synthesis allows for rapid mobilization of energy (Platzer et al., 1999). IJs are the only free-living stage in *Steinernema* life cycle and may be exposed to changes in oxygen content in the soil environment. A FT-NMR spectroscopy study also demonstrated that the production of ADP catalyzed by arginine kinase in *S. carpocapsae*, suggesting the level of phosphoarginine may be an indicator of anaerobiosis and have a pivotal role in the regulation of the rate of anaerobic ATP synthesis (through reduction of fumarate to succinate; Thompson et al., 1992).

Here, we showed evidence that transcripts involved in the arginine pathway are downregulated in both *S. carpocasae* and *S. puntauvense* IJs that were *in vitro* reared (either colonized or aposymbiotic). We speculate these genes may be involved in the energy metabolism and ROS homeostasis of IJs thus affects their longevity.

These observations could also be correlated with the different host foraging behaviors the studies *Steinernema* species have. While *S. carpocapsae* IJs are ambushers (Campbell and Gaugler, 1993), *S. puntauvense* has an intermediate host seeking behavior that combines ambushing and cruising (P. Stock pers. comm). In relation to this, we hypothesize that energy requirements of IJs may be different and can be correlated with differences observed in the transcriptomic analysis including a stronger down-regulation of metabolism pathways for *S. puntauvense* in absence of an insect host when compared with *S. carpocapsae*.

### Absence of the *Xenorhabdus* symbiont Causes Upheaval of Venom Protein Expression

Numerous studies have demonstrated that *Xenorhabdus* symbionts play a key role in aiding *Steinernema* IJs invade insect host by producing toxins, virulence factors and secondary metabolites (Crawford et al., 2012; Eleftherianos et al., 2018; Shi and Bode, 2018). When, IJs invade insect host, the release of *Xenorhabdus* is not immediate and the nematodes need to escape to host’s encapsulation and melanization response (Wang et al., 1994). In this respect, Walter et al. (2008) showed that *S. carpocapsae* IJs can release metabolites that inhibit haemocytic encapsulation in the insect host. Recently, it was demonstrated venom proteins are released when IJs initiate active parasitism (Lu et al., 2017; Chang et al., 2019). This venom proteins display toxicity in several insect hosts even when harvested from aposymbiotic nematodes (Lu et al., 2017). Furthermore, it has been suggested that both the nematodes and *Xenorhabdus* symbiont contribute to the insect host invasion *via* these proteins (Lu et al., 2017). Interestingly, our transcriptomic analysis showed a strong differential expression of transcripts homologous to venom proteins in IJs depleted of *Xenorhabdus*, suggesting an adaptation by the nematodes to compensate for the absence of their symbiotic partners.

Among the abundant venom proteins, we observed a general down-regulation of the ubiquitin family for both species, in nematodes reared in the absence of their symbiont. The ubiquitin is a family comprised a group of highly conserved 76-amino acid polypeptide proteins known specific to eukaryotes which bind to the amino groups of the target protein *via* its C-terminal glycine (Pickart and Eddins, 2004). In *C. elegans*, ubiquitin proteins appear involved in mechanisms such as the regulation of immune signaling (Garcia-Sanchez et al., 2021). In the plant parasitic nematode, *Heterodera schachtii*, also known as the cyst nematode, it has been shown that ubiquitin protein is secreted by the dorsal pharyngeal gland and it is highly expressed during initial infection stages [78] This protein may also have a regulatory role in cell formation of the cysts in the host plant (Tytgat et al., 2004). Based on results from the present study, we speculate that presence of *Xenorhabdus* symbionts may be required to induce immune mechanisms triggered by ubiquitin protein.

Here, we shown contrasting results for expression of trypsin-like serine protease (TrypSPc) and the trypsin inhibitor (TIL) identified as venom proteins, when comparing the two studied *Steinernema* spp. Specifically, we observed up-regulation of both proteins in absence of symbiont in *S. carpocapsae* IJs, whereas a down-regulation of the TIL protein was denoted in *S. puntauvense*. In general, serine protease activity has been reported in numerous parasitic nematodes and appears to be involved in a wide variety of events in the life cycle (such as molting, nutrition and host invasion; Yang et al., 2015). It has been demonstrated that trypsin serine protease purified from excreted-secreted products of *S. carpocapsae* IJs has the ability to prevent the insect’s haemocytes from spreading, and it also alters haemocytes cytoskeleton (Balasubramanian et al., 2010). Thus, these serine proteases prevent melanotic encapsulation, one important mechanism of insect defense against nematodes. The effect of other serine proteases, such as chymotrypsin serine protease Sc-SP-1 is potentially involved in immune host evasion (Toubarro et al., 2010) and the elastase serine protease Sc-ELA potentially associated with developmental and fibrinolytic activities, has also been investigated in *S. carpocapsae* (Hao et al., 2009). Serine proteases are actively involved in host–parasite interactions but these relationships appear very specific (Yang et al., 2015). Our study suggests that the absence of symbiont in *S. carpocapsae* IJs increases expression of many trypsin serine proteases. Conversely, the expression of the trypsin inhibitor domain is decreased in *S. puntauvense* in absence of an insect host (both *in vitro* colonized and aposymbiotic). In this respect, we speculate that this putative mechanism of host evasion based on trypsin-like serine protease might be specific to *S. carpocapsae*. Contrasting results among the two *Steinernema species* were also observed for the expression of serine carboxypeptidase. For both species, a differential expression is observed in the absence of symbiont in both species. However, a differential regulation was observed between *in vitro* colonized nematodes and *in vitro* aposymbiotic nematodes in both species. For example, the serine carboxypeptidase transcript homologous to L596_001158 (orthogroup OG286) was downregulated in the *in vitro* colonized nematodes for both species, but it was upregulated in absence of symbiont for *S. carpocapsae* and down-regulated for *S. puntauvense*, The serine carboxypeptidases (SCPs) were described as involved in numerous physiological processes ranging from digestion to the biosynthesis of peptides that function in cell–cell signaling (Fricker, 2007). Recently, it has been suggested that the SCPs could contribute to parasitism in nematodes. For example, in the plant-parasitic nematode *Radopholus similis*, RNA interference (RNAi) of Rs-scp-1 reduced its virulence (Huang et al., 2017). Additionally, in the entomopathogenic nematodes *Heterorhabditis bacteriophora*, SCP Hb-sc-1 exhibited both toxic and immunomodulation properties in *Drosophila* (Kenney et al., 2021). In this respect, our data suggest regulation of serine carboxypeptidases in absence of symbiont and/or in absence of insect host depending on the individual *Steinernema* species tested and that this may be correlated with their respective differential pathogenic activities.

Another interesting result from this study is the differential expression of genes encoding the fatty acid retinoid binding proteins (FAR), which have been shown to be secreted by the IJs. Differential expression of the analyzed FAR proteins was observed for both species in absence of the symbiont, with a subtle tendency of up-regulation (six upregulated and five downregulated for *S. carpocapsae*; seven upregulated and four downregulated for *S. puntauvense*).

FAR proteins have been described as unique proteins specific to nematodes (Garofalo et al., 2003). Although, recently horizontal transfer of FAR genes in bacteria genomes has been suggested within *Kitasatospora*, *Bacillus*, *Lysobacter*, and *Streptomyces* (Yuan et al., 2021). The FAR proteins are intriguing and despite early investigations on their ligand-binding propriety, very little is known regarding their function *in vivo* (Garofalo et al., 2003). It has been suggested that the FAR proteins might play role in the binding lipids from their environment or host (Kennedy et al., 2013). The impact of these proteins, in particular FAR-1, on reproduction was recently demonstrated in the plant-parasitic *Pratylenchus penetrans* using RNAi (Vieira et al., 2017). Additionally, in *Globodera pallida*, Gp-FAR-1 appears to bind to lipids precursors of plant defense mechanisms suggesting manipulation of the lipid-based signaling pathway (Prior et al., 2001). The FAR proteins have been identified in numerous nematodes genomes and that their numbers are highly variable. For example, eight were identified in *C. elegans* genome and 19 in *Pristionchus pacificus* genome, and in parasitic-nematodes, eight in *Necator americanus*, 1–4 in *Globodera*, 18–30 in *Ancylostoma* (Garofalo et al., 2003; Dieterich et al., 2008; Yuan et al., 2021).

A previous genome study by Dillman et al. (2015) showed an exhaustive expansion of the FAR genes in *Steinernema*, where between 38 and 54 were identified. A high expression of the some FAR genes during L1 stages and other FAR genes was observed in IJs suggesting their involvement in development and parasitism (Dillman et al., 2015). The FAR genes were identified in abundance in both excreted proteins in *S. carpocapsae* and *S. feltiae* (Lu et al., 2017; Chang et al., 2019). In this respect, our results showed FAR genes expression is modified in absence of the symbiont for both species. We speculate this may an adaptation of *Steinernema* IJs to achieve host invasion in the absence of their symbiotic partners.

In summary, we observed the regulation of transcripts belonging to various genes, including FAR proteins, ubiquitin, trypsin-like serine protease or serine carboxypeptidases in aposymbiotic IJs. We predict they may play a role in the nematodes’ host invasion mechanisms, in the absence of their symbiont. Previous studies examined the fitness of *Steinernema* IJs in the absence of *Xenorhabdus* symbiont (Sicard et al., 2003; McMullen et al., 2017). These studies showed that in absence of *X. nematophila*, *Steinernema carpocapsae* IJs have a decreased infection success (80%–46%) and progeny production (seven times less progeny; Sicard et al., 2003). However, in *S. puntauvense*, aposymbiotic IJs maintain their virulence although their ability to produced IJs progeny was compromised suggesting a stronger disruption in their reproductive fitness than that observed in *S. carpocapsae* [17]. Here, we denoted contrasting variation in the expression of venom proteins in the two tested *Steinernema* species, suggesting that the mechanisms involved in the production and regulation of these proteins may be species-specific. In addition, our results showed that *in vitro* rearing appears to affect the expression of the venom proteins. This subject should be further explored in the context of *in vitro* mass-production of *Steinenerma* nematodes (Saleh et al., 2019). However, our study used the liver-kidney agar plates which are not the major medium used for mass production of EPNs, so similar study comparing metabolic costs by RNA-seq approach of different rearing methods appears very promising. Further investigations are warranted to expand on this topic.

## Data Availability Statement

The datasets presented in this study can be found in online repositories. The names of the repository/repositories and accession number(s) can be found at: BioProject PRJNA766056; Biosample SAMN21601377, SAMN21601527, SAMN21601552 SAMN21604292, SAMN21604906, SAMN21604907; SRA study SRP338612 (SRR16057604 to SRR16057609); Transcriptome Shotgun Assembly project GJLD00000000 and GJLE00000000; GEO accession GSE185177.

## Author Contributions

SS conceived, designed the experiments, and supervised. EL and JM performed the experiments. EL investigated and analyzed the data. EL and SS wrote the main manuscript text. All authors contributed to the article and approved the submitted version.

## Conflict of Interest

The authors declare that the research was conducted in the absence of any commercial or financial relationships that could be construed as a potential conflict of interest.

## Publisher’s Note

All claims expressed in this article are solely those of the authors and do not necessarily represent those of their affiliated organizations, or those of the publisher, the editors and the reviewers. Any product that may be evaluated in this article, or claim that may be made by its manufacturer, is not guaranteed or endorsed by the publisher.

## Footnotes

^1^ https://github.com/FelixKrueger/TrimGalore

^2^ https://github.com/s-andrews/FastQC

^3^ http://transdecoder.github.io/

^4^ https://inp.univ-amu.fr/en/

## Supplementary Material

Supplementary Figure S1 Barplot of enriched KEGG ratio of differentially expressed transcript in the *in vitro* colonized reared nematodes compare to *in vivo* reared nematodes using i-KOBAS. The length of the bar represents the enrich ratio calculated as “input gene number”/ “backgound gene number.” The color of the bar represent network of protein representing modules identified by i-KOBAS based on *C. elegans* dataset. For network that numerous modules are enriched only the top 5 is displayed. The Asterix indicated enriched KEGG term identify regulated in the same type of analysis on in the *in vitro* aposymbiotic reared nematodes. **(A)** Enriched KEGG ration of upregulated transcripts. **(B)** Enriched KEGG ratio of downregulated transcripts.

Supplementary Figure S2 Chart pie of differentially expressed transcripts in the *in vitro* aposymbiotic reared nematodes compare to *in vivo* reared nematodes classified by KEGG categories identify by i-KOBAS. For each species, two chart pies were produced, one for the downregulated transcripts and the other for the upregulated transcripts. **(A)** The differentially expressed transcripts are classified into four categories: metabolism pathway, genetic information processing, environmental information processing and cellular processes. **(B)** The differentially expressed transcripts are classified into 19 KEGG terms.

Supplementary Table S1 Information on the custom protein database used to filter transcriptomic assemblies.

Supplementary Table S2 Information about the design and optimization of primers for qRT-PCR validation.

Supplementary Table S3 Information on reads mapping to the *Xenorhabdus* genomes references. The reads were mapped to the reference using Tophat. Coverage statistics was established using shell script based on samtools.

Supplementary Table S4 Enriched KEGG pathway of differentially expressed transcripts in the *in vitro* aposymbiotic condition compared to the *in vivo* condition using i-KOBAS classified by KEGG categories.

Supplementary Table S5 Summary of statistically analyses performed in the study. Statistical tests on the relative expression established by qRT-PCR for both species are summarized, as well as the tests on the glycogen concentration of *S. puntauvense* IJs. All the tests were performed in the R environment. For all the data, we tested if the data were normally distributed (Shapiro–Wilk normality test), present common variance (Levene’s test) and were independent group (Chi-squared test). If these three assumptions are validated, the variance was analyzed using one way ANOVA associated with post-hoc Tukey HSD to compare the three conditions. If not, the non-parametric Kruskal-wallis rank sum associated with multiple pairwise comparisons using the dunn’s test was used.

Supplementary Table S6 Information on transcript homologues to S. carpocapsae venom proteins. For each transcript identify as homologues to venom proteins is indicated: name of the transcript; the name of the protein; annotation based on hmmer; the homologous protein from the *S. carpocapsae* reference; summary of differential expression and detail based on cufflinks and edgeR; raw gene counts (the two replicates). Abbreviation: VV: *in vivo* condition versus *in vitro* colonized condition; VA: *in vivo* condition versus *in vitro* aposymbiotic; D: Downregulated; U: Upregulated; NS: nonsignificant.
