# Supplementary figures and images for: Transcriptomic Analysis of Steinernema Nematodes Highlights Metabolic Costs Associated to Xenorhabdus Endosymbiont Association and Rearing Conditions
Source: Front Physiol. 2022 Feb 25;13:821845. doi: 10.3389/fphys.2022.821845 (PMC8914265; doi:10.3389/fphys.2022.821845)

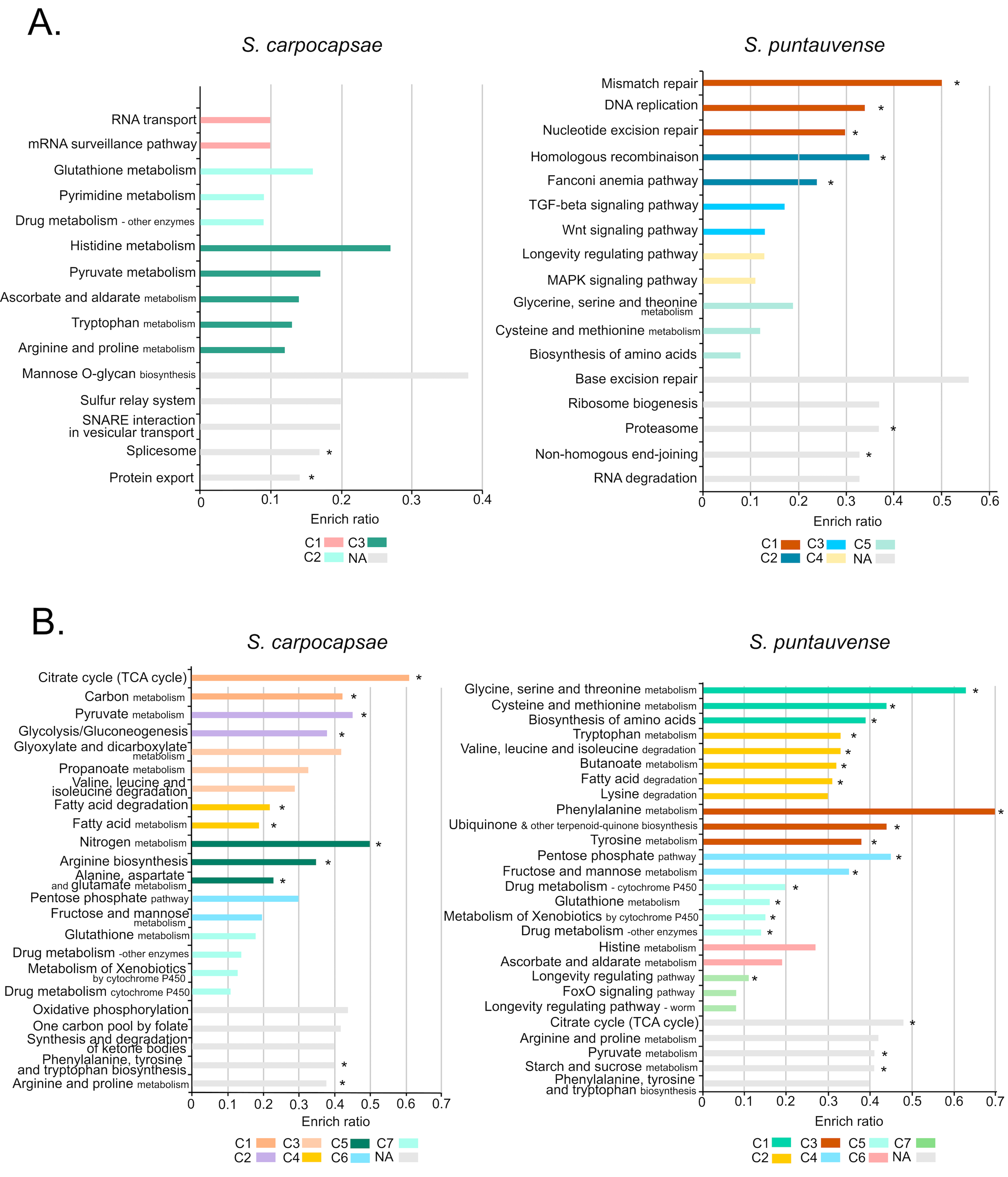

Supplement: Supplementary Figure S1 — Barplot of enriched KEGG ratio of differentially expressed transcript in the in vitro colonized reared nematodes compare to in vivo reared nematodes using i-KOBAS. The length of the bar represents the enrich ratio calculated as “input gene number”/ “backgound gene number.” The color of the bar represent network of protein representing modules identified by i-KOBAS based on C. elegans dataset. For network that numerous modules are enriched only the top 5 is displayed. The Asterix indicated enriched KEGG term identify regulated in the same type of analysis on in the in vitro aposymbiotic reared nematodes. (A) Enriched KEGG ration of upregulated transcripts. (B) Enriched KEGG ratio of downregulated transcripts. [file Data_Sheet_1.ZIP › S1Fig.tif]

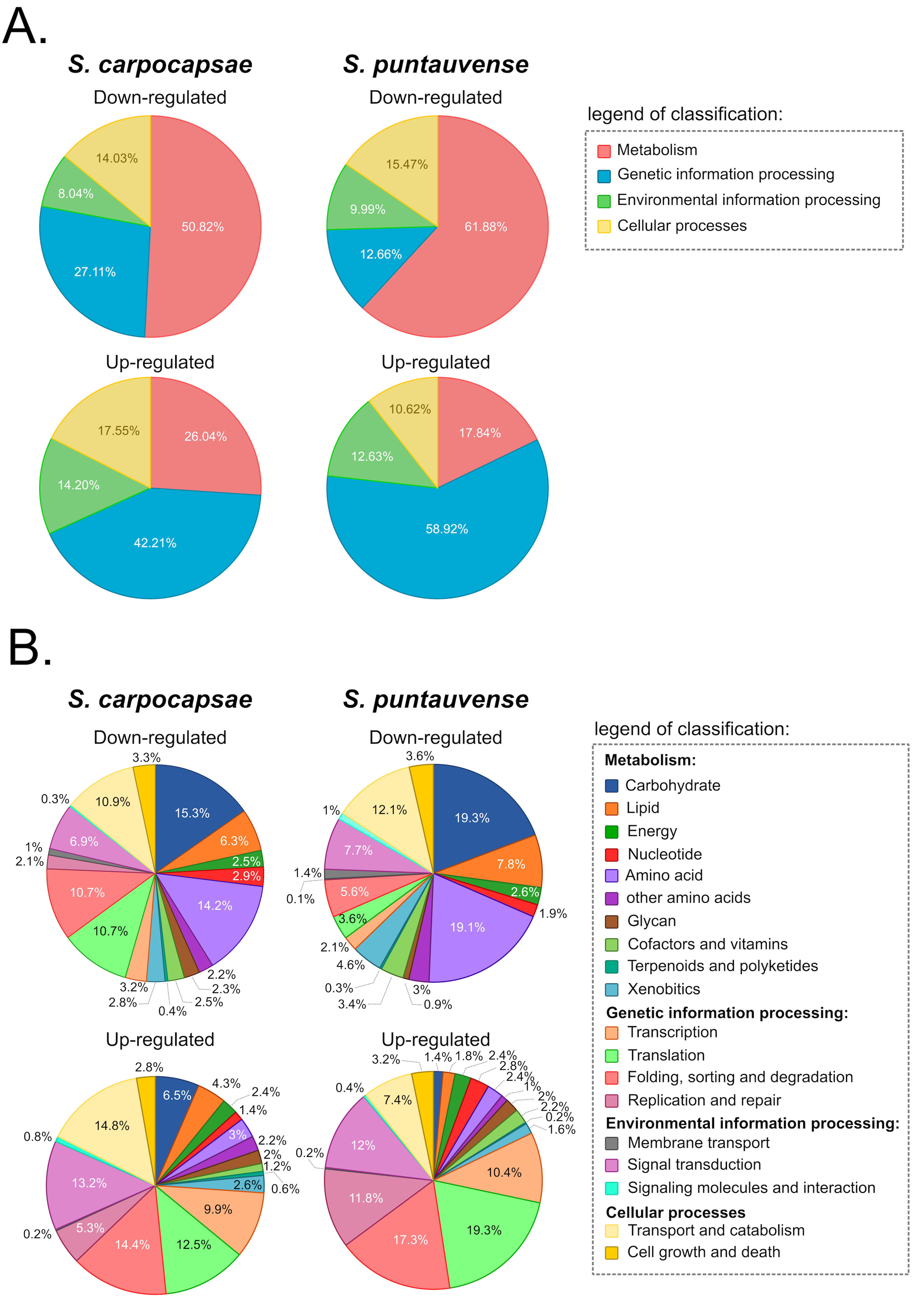

Supplement: Supplementary Figure S1 — Barplot of enriched KEGG ratio of differentially expressed transcript in the in vitro colonized reared nematodes compare to in vivo reared nematodes using i-KOBAS. The length of the bar represents the enrich ratio calculated as “input gene number”/ “backgound gene number.” The color of the bar represent network of protein representing modules identified by i-KOBAS based on C. elegans dataset. For network that numerous modules are enriched only the top 5 is displayed. The Asterix indicated enriched KEGG term identify regulated in the same type of analysis on in the in vitro aposymbiotic reared nematodes. (A) Enriched KEGG ration of upregulated transcripts. (B) Enriched KEGG ratio of downregulated transcripts. [file Data_Sheet_1.ZIP › S2Fig.tif]
